# Correction to: How visual and proprioceptive feedback mediate the effect of monetary incentive on motor precision

**DOI:** 10.3758/s13414-025-03143-1

**Published:** 2025-08-04

**Authors:** Nicholas Menghi, Giorgio Coricelli, Clayton Hickey

**Affiliations:** 1 Department of Psychology, Max Planck for Human Cognitive and Brain Sciences, Leipzig, Germany; 2 Department of Economics, University of Southern California, Los Angeles, USA; 3 Laboratory for the Psychology of Child Development (LaPsyDÉ), UMR CNRS 8240, Paris, France; 4 Centre for Human Brain Health and School of Psychology, University of Birmingham, Birmingham, UK

**Correction to: Attention, Perception, & Psychophysics**

10.3758/s13414-025-03132-4

The author name "Georgia Coricelli" should be corrected to "Giorgio Coricelli."

The original article has been corrected.

