# Supplementary material for: How visual and proprioceptive feedback mediate the effect of monetary incentive on motor precision
Source: Atten Percept Psychophys. 2025 Jul 23;87(8):2488–503. doi: 10.3758/s13414-025-03132-4 (PMC12568819; doi:10.3758/s13414-025-03132-4)
Supplement: Supplementary file 1 — Supplementary file1 (PDF 1906 KB) [file 13414_2025_3132_MOESM1_ESM.pdf]

Attention, Perception,
& Psychophysics

How visual and proprioceptive feedback mediate the effect of monetary incentive on motor precision

Journal:	*Attention, Perception, & Psychophysics*
Manuscript ID	PP-ORIG-24-271.R1
Manuscript Type:	Original Manuscript
Date Submitted by the Author:	02-Mar-2025
Complete List of Authors:	Menghi, Nicholas; Max Planck Institute for Human Cognitive and Brain Sciences, Department of Psychology<br>Coricelli, Giorgio; University of Southern California, Department of Economics; CNRS, Laboratory for the Psychology of Child Development (LaPsyDÉ)<br>Hickey, Clayton; University of Birmingham, Centre for Human Brain Health and School of Psychology
Keywords:	motor control, Perception and Action, Multisensory Processing

SCHOLARONE™
Manuscripts

Supplementary Materials

In the review process, an anonymous reviewer requested that we provide results from the set of possible *post hoc* contrasts that are motivated by significant ANOVA effects described in the body of the paper. These are provided below.

1.1 Experiment 1 - Initial force estimation

Pairwise comparison of the Exertion main effect

Exertion	Exertion	Difference	StdErr	p-value	Lower	Upper
**38**	46	2.18	0.36	<0.001	1.096	3.264
**38**	52	5.777	0.605	<0.001	3.956	7.597
**38**	63	9.106	0.895	<0.001	6.416	11.796
**38**	70	10.953	1.613	<0.001	6.103	15.803
**46**	52	3.597	0.411	<0.001	2.362	4.831
**46**	63	6.926	0.661	<0.001	4.938	8.914
**46**	70	8.773	1.362	<0.001	4.678	12.868
**52**	63	3.33	0.401	<0.001	2.125	4.534
**52**	70	5.177	1.115	0.001	1.823	8.53
**63**	70	1.847	0.898	0.278	-0.854	4.548

SM Table 1. Multiple comparisons of force estimation error across exertion levels. Pairwise comparisons were conducted using Tukey's HSD test to examine the effects of exertion on force estimation error. The table presents the mean difference in estimation error between exertion levels, along with the standard error (StdErr), p-value, and the 95% confidence interval (Lower and Upper bounds). Significant comparisons ($p < 0.05$) indicate that force estimation error increased with higher exertion levels, except for the comparison between 63% and 70% exertion, which did not reach significance.

Pairwise comparison of the Exertion by Feedback Interaction

Feedback	Exertion	Exertion	Difference	StdErr	p-value	Lower	Upper
**No**	38	46	3.813	0.653	<0.001	1.849	5.776
**No**	38	52	9.241	1.036	<0.001	6.125	12.357
**No**	38	63	14.292	1.36	<0.001	10.201	18.382
**No**	38	70	15.041	2.484	<0.001	7.57	22.512
**No**	46	52	5.428	0.62	<0.001	3.564	7.292
**No**	46	63	10.479	0.92	<0.001	7.713	13.245
**No**	46	70	11.228	2.011	<0.001	5.181	17.276
**No**	52	63	5.051	0.641	<0.001	3.123	6.978
**No**	52	70	5.8	1.631	0.016	0.895	10.706
**No**	63	70	0.749	1.399	0.982	-3.456	4.955
**yes**	38	46	0.548	0.171	0.034	0.033	1.062
**yes**	38	52	2.312	0.359	<0.001	1.234	3.391
**yes**	38	63	3.921	0.637	<0.001	2.004	5.838

yes	38	70	6.865	1.086	<0.001	3.6	10.13
yes	46	52	1.765	0.372	0.001	0.645	2.885
yes	46	63	3.373	0.616	<0.001	1.521	5.225
yes	46	70	6.318	1.03	<0.001	3.221	9.415
yes	52	63	1.608	0.484	0.026	0.153	3.064
yes	52	70	4.553	0.859	<0.001	1.971	7.135
yes	63	70	2.945	0.732	0.006	0.742	5.147

SM Table 2. Multiple comparisons of force estimation error across exertion levels in different feedback conditions. Pairwise comparisons using Tukey's HSD test examined the effect of exertion on force estimation error separately for the no-feedback and feedback conditions. The table presents the mean difference in estimation error between exertion levels, along with the standard error (StdErr), p-value, and the 95% confidence interval (Lower and Upper bounds). Results indicate that force estimation error increased with exertion in both feedback conditions, though the differences were generally more pronounced in the no-feedback condition. Comparisons that did not reach significance are also reported.

Post hoc analyses of the three-way ANOVA

To further investigate the three-way interaction, we conducted separate two-way repeated measures ANOVAs for each of the feedback and no-feedback conditions separately.

In the no feedback condition, the analysis revealed a significant main effect of exertion, $F(4,76) = 43.153$, $p < 0.001$, indicating that force estimation error varied across exertion levels. Neither the main effect of incentive, $F(1,19) = 2.900$, $p = 0.105$, nor the interaction between exertion and incentive were significant,, $F(4,76) = 0.397$, $p = 0.810$. In the total feedback condition, a significant main effect of exertion emerged, $F(4,76) = 32.000$, $p < 0.001$, indicating that force estimation error varied across exertion levels. A significant main effect of incentive was also found, $F(1,19) = 5.411$, $p = 0.031$, suggesting that incentives influenced force estimation error. Additionally, a significant interaction between exertion and incentive was observed, $F(4,76) = 3.758$, $p = 0.008$, indicating that the effect of incentives on force estimation error depended on exertion levels.

Assessing the robustness of the Exertion by Incentive difference by performing ANOVA after excluding a participant with a large effect.

Results illustrated in Figure 3 Panel C suggest the possibility that results from a single participant may have contributed substantially to the statistical result (even though this observation did not meet our criteria for outlier results).

To probe this, we repeated this analysis employing data that excluded this observation. The analysis confirmed the presence of both main effects and their interaction (Exertion: $F = 29.224$, $p < 0.001$; Incentive: $F = 4.774$, $p = 0.042$; Exertion × Incentive: $F = 2.561$, $p = 0.045$).

1.2 Experiment 1 - Consistency Across Trials

Pairwise comparison of the Exertion main effect

Exertion	Exertion	Difference	StdErr	p-value	Lower	Upper
**38**	46	-0.103	0.28	0.996	-0.946	0.74
**38**	52	-1.217	0.472	0.115	-2.636	0.203
**38**	63	-1.929	0.475	0.005	-3.357	-0.5
**38**	70	-4.854	0.727	<0.001	-7.039	-2.67
**46**	52	-1.114	0.319	0.018	-2.074	-0.154
**46**	63	-1.826	0.44	0.004	-3.15	-0.502
**46**	70	-4.752	0.598	<0.001	-6.55	-2.953
**52**	63	-0.712	0.462	0.549	-2.101	0.676
**52**	70	-3.638	0.436	<0.001	-4.949	-2.326
**63**	70	-2.926	0.594	0.001	-4.711	-1.14

SM Table 3. Results of post-hoc comparisons examining the effect of exertion on force estimation error. The table presents pairwise differences between exertion levels (38% - 70% MVC), with standard errors, p-values, and confidence intervals (lower and upper bounds). Significant comparisons ($p < 0.05$) indicate a change in force estimation error between exertion levels.

Pairwise comparison of the Exertion by Incentive Interaction

Incentive	Exertion	Exertion	Difference	StdErr	p-value	Lower	Upper
**High**	38	46	-0.189	0.37	0.985	-1.301	0.923
**High**	38	52	-0.745	0.468	0.519	-2.151	0.661
**High**	38	63	-1.502	0.521	0.064	-3.067	0.064
**High**	38	70	-3.802	0.766	0.001	-6.104	-1.5
**High**	46	52	-0.556	0.346	0.51	-1.595	0.483
**High**	46	63	-1.313	0.543	0.153	-2.945	0.32
**High**	46	70	-3.613	0.624	<0.001	-5.491	-1.735
**High**	52	63	-0.757	0.509	0.584	-2.289	0.775
**High**	52	70	-3.057	0.478	<0.001	-4.494	-1.62
**High**	63	70	-2.3	0.746	0.043	-4.542	-0.058
**Low**	38	46	-0.016	0.315	1	-0.963	0.93
**Low**	38	52	-1.688	0.684	0.14	-3.745	0.368
**Low**	38	63	-2.356	0.645	0.013	-4.296	-0.415
**Low**	38	70	-5.907	0.874	<0.001	-8.534	-3.279
**Low**	46	52	-1.672	0.548	0.046	-3.319	-0.025
**Low**	46	63	-2.34	0.615	0.009	-4.19	-0.489
**Low**	46	70	-5.89	0.751	<0.001	-8.148	-3.633
**Low**	52	63	-0.667	0.751	0.898	-2.925	1.591

**Low**	52	70	-4.218	0.55	<0.001	-5.872	-2.564
**Low**	63	70	-3.551	0.81	0.003	-5.986	-1.116

SM Table 4. Results of post-hoc comparisons examining the interaction between exertion and incentive on force estimation error. The table presents pairwise differences between exertion levels (38% - 70% MVC) separately for high and low incentive conditions, along with standard errors, p-values, and confidence intervals (lower and upper bounds). Significant comparisons ($p < 0.05$) suggest an effect of exertion within each incentive condition.

1.3 Experiment 1 - Sustained Force Maintenance

Pairwise comparison of the Exertion Main effect

Exertion	Exertion	Difference	StdErr	p-value	Lower	Upper
**38**	46	1.773	0.282	<0.001	0.924	2.621
**38**	52	4.701	0.462	<0.001	3.313	6.09
**38**	63	7.884	0.737	<0.001	5.669	10.099
**38**	70	8.57	1.358	<0.001	4.486	12.655
**46**	52	2.929	0.315	<0.001	1.98	3.877
**46**	63	6.111	0.589	<0.001	4.339	7.884
**46**	70	6.798	1.181	<0.001	3.245	10.35
**52**	63	3.183	0.399	<0.001	1.982	4.384
**52**	70	3.869	1.033	0.011	0.763	6.976
**63**	70	0.687	0.774	0.898	-1.642	3.015

SM Table 5. Results of post-hoc comparisons for exertion levels (38% - 70% MVC) on force estimation error. The table presents pairwise differences between exertion levels, including standard errors, p-values, and confidence intervals (lower and upper bounds). Significant comparisons ($p < 0.05$) indicate an effect of exertion on force estimation error.

Pairwise comparison of the Exertion by Feedback Interaction

Feedback	Exertion	Exertion	Difference	StdErr	p-value	Lower	Upper
**No**	38	46	3.207	0.567	<0.001	1.504	4.911
**No**	38	52	8.508	0.913	<0.001	5.764	11.253
**No**	38	63	13.402	1.237	<0.001	9.681	17.123
**No**	38	70	12.479	2.174	<0.001	5.941	19.017
**No**	46	52	5.301	0.638	<0.001	3.383	7.218
**No**	46	63	10.195	0.94	<0.001	7.368	13.021
**No**	46	70	9.272	1.793	<0.001	3.881	14.663
**No**	52	63	4.894	0.598	<0.001	3.094	6.693
**No**	52	70	3.971	1.489	0.097	-0.506	8.448

**No**	63	70	-0.923	1.239	0.943	-4.648	2.803
**yes**	38	46	0.338	0.068	0.001	0.134	0.542
**yes**	38	52	0.894	0.16	<0.001	0.413	1.375
**yes**	38	63	2.366	0.452	<0.001	1.007	3.724
**yes**	38	70	4.662	1.062	0.003	1.468	7.855
**yes**	46	52	0.556	0.134	0.005	0.152	0.961
**yes**	46	63	2.028	0.439	0.002	0.708	3.348
**yes**	46	70	4.324	1.052	0.005	1.16	7.487
**yes**	52	63	1.471	0.351	0.004	0.415	2.528
**yes**	52	70	3.767	0.942	0.006	0.935	6.6
**yes**	63	70	2.296	0.71	0.031	0.161	4.431

SM Table 6. Post-hoc comparisons for exertion levels (38% - 70% MVC) on force estimation error, separated by feedback condition (No vs. Total). The table presents pairwise differences between exertion levels, including standard errors, p-values, and confidence intervals (lower and upper bounds). Significant comparisons ($p < 0.05$) suggest an effect of exertion on force estimation error within each feedback condition.

Post hoc analyses of the three-way interaction

Furthermore, to further investigate the three-way interaction, we conducted two, two-way repeated measures ANOVA looking for the effect of incentive.

In the no feedback condition, the analysis revealed a significant main effect of exertion, $F(4,76) = 42.223$, $p < 0.001$, indicating that force estimation error varied across exertion levels. The main effect of incentive was not significant, $F(1,19) = 4.153$, $p = 0.055$. Similarly, the interaction between exertion and incentive did not reach significance, $F(4,76) = 0.934$, $p = 0.448$. In the total feedback condition, we found a significant main effect of exertion, $F(4,76) = 14.4$, $p < 0.001$, indicating that force estimation error varied across exertion levels. A significant main effect of incentive was also found, $F(1,19) = 4.3868$, $p = 0.049$, suggesting that incentives influenced force estimation error. Additionally, a significant interaction between exertion and incentive was observed, $F(4,76) = 3.8692$, $p = 0.008$, indicating that the effect of incentives on force estimation error depended on exertion levels.

1.4 Experiment 1 - Deviation within trials in Force Maintenance

Pairwise comparison of the Exertion Main Effect

Exertion	Exertion	Difference	StdErr	p-value	Lower	Upper
**38**	46	-0.163	0.092	0.414	-0.439	0.113
**38**	52	-0.319	0.115	0.08	-0.666	0.027
**38**	63	-0.863	0.192	0.002	-1.441	-0.286
**38**	70	-1.487	0.271	<0.001	-2.303	-0.671
**46**	52	-0.156	0.085	0.379	-0.411	0.099
**46**	63	-0.7	0.16	0.003	-1.182	-0.218

**46**	70	-1.324	0.246	<0.001	-2.063	-0.586
**52**	63	-0.544	0.124	0.003	-0.918	-0.17
**52**	70	-1.168	0.205	<0.001	-1.784	-0.551
**63**	70	-0.624	0.192	0.031	-1.202	-0.046

SM Table 7. Pairwise comparisons of exertion levels (38% - 70% MVC) on deviation within trials. The table presents the mean difference between exertion levels, standard errors (StdErr), p-values, and 95% confidence intervals (lower and upper bounds). Significant comparisons ($p < 0.05$) indicate a systematic change in deviations with increasing exertion levels.

Pairwise comparison of the Exertion by Feedback Interaction

Feedback	Exertion	Exertion	Difference	StdErr	p value	Lower	Upper
**No**	38	46	-0.065	0.149	0.992	-0.514	0.383
**No**	38	52	-0.158	0.173	0.889	-0.679	0.363
**No**	38	63	-0.38	0.173	0.225	-0.901	0.141
**No**	38	70	-0.762	0.254	0.051	-1.525	0.002
**No**	46	52	-0.093	0.144	0.966	-0.526	0.34
**No**	46	63	-0.315	0.153	0.28	-0.775	0.146
**No**	46	70	-0.697	0.241	0.063	-1.422	0.028
**No**	52	63	-0.222	0.105	0.252	-0.537	0.093
**No**	52	70	-0.604	0.148	0.005	-1.05	-0.159
**No**	63	70	-0.382	0.171	0.21	-0.896	0.132
**yes**	38	46	-0.261	0.079	0.026	-0.498	-0.024
**yes**	38	52	-0.481	0.085	<0.001	-0.736	-0.226
**yes**	38	63	-1.347	0.237	<0.001	-2.061	-0.633
**yes**	38	70	-2.213	0.411	<0.001	-3.449	-0.977
**yes**	46	52	-0.22	0.078	0.075	-0.456	0.016
**yes**	46	63	-1.086	0.23	0.001	-1.779	-0.393
**yes**	46	70	-1.952	0.392	0.001	-3.13	-0.773
**yes**	52	63	-0.866	0.196	0.002	-1.454	-0.277
**yes**	52	70	-1.732	0.369	0.001	-2.842	-0.621
**yes**	63	70	-0.866	0.354	0.145	-1.929	0.198

SM Table 8. Pairwise comparisons of exertion levels (38% - 70% MVC) on deviation within trials, separated by feedback conditions. The table presents the mean difference between exertion levels, standard errors (StdErr), p-values, and 95% confidence intervals (lower and upper bounds). Significant comparisons ($p < 0.05$) indicate systematic differences in force deviation across exertion levels, with feedback modulating the effects.

Pairwise comparison of the Exertion by Incentive Interaction

Incentive	Exertion	Exertion	Difference	StdErr	p value	Lower	Upper
**High**	38	46	-0.146	0.13	0.791	-0.536	0.244
**High**	38	52	-0.281	0.135	0.27	-0.687	0.126
**High**	38	63	-0.691	0.187	0.012	-1.253	-0.128
**High**	38	70	-1.073	0.247	0.003	-1.815	-0.332
**High**	46	52	-0.135	0.154	0.902	-0.598	0.328
**High**	46	63	-0.545	0.172	0.036	-1.061	-0.029
**High**	46	70	-0.928	0.219	0.004	-1.586	-0.269
**High**	52	63	-0.41	0.092	0.002	-0.686	-0.133
**High**	52	70	-0.793	0.191	0.004	-1.366	-0.219
**High**	63	70	-0.383	0.169	0.197	-0.89	0.124
**Low**	38	46	-0.18	0.128	0.63	-0.566	0.205
**Low**	38	52	-0.358	0.143	0.133	-0.789	0.073
**Low**	38	63	-1.036	0.253	0.005	-1.796	-0.276
**Low**	38	70	-1.901	0.378	0.001	-3.037	-0.765
**Low**	46	52	-0.177	0.119	0.583	-0.537	0.182
**Low**	46	63	-0.856	0.238	0.015	-1.573	-0.139
**Low**	46	70	-1.721	0.37	0.001	-2.834	-0.607
**Low**	52	63	-0.678	0.208	0.03	-1.304	-0.052
**Low**	52	70	-1.543	0.353	0.003	-2.604	-0.482
**Low**	63	70	-0.865	0.346	0.132	-1.905	0.175

SM Table 9. Pairwise comparisons of exertion levels (38% - 70% MVC) on deviation within trials, separated by incentive conditions (High/Low). The table presents the mean difference between exertion levels, standard errors (StdErr), p-values, and 95% confidence intervals (lower and upper bounds). Significant comparisons ($p < 0.05$) suggest systematic differences in deviations across exertion levels, with incentive levels modulating these effects.

1.5 Experiment 1 - Consistency within trials in Force Maintenance

Pairwise comparison of the Exertion Main Effect

Exertion	Exertion	Difference	StdErr	p value	Lower	Upper
**38**	46	0.162	0.132	0.739	-0.236	0.56
**38**	52	-0.267	0.263	0.846	-1.059	0.525
**38**	63	-0.793	0.292	0.09	-1.672	0.087
**38**	70	-1.734	0.351	0.001	-2.789	-0.678

**46**	52	-0.429	0.208	0.276	-1.054	0.196
**46**	63	-0.955	0.274	0.018	-1.777	-0.132
**46**	70	-1.896	0.287	<0.001	-2.758	-1.033
**52**	63	-0.526	0.261	0.296	-1.31	0.258
**52**	70	-1.467	0.235	<0.001	-2.175	-0.759
**63**	70	-0.941	0.288	0.029	-1.807	-0.075

SM Table 10. Pairwise comparisons of exertion levels (38% - 70% MVC) on consistency. The table presents the mean differences between exertion levels, standard errors (StdErr), p-values, and 95% confidence intervals (lower and upper bounds). Significant differences (p < 0.05) indicate systematic changes in consistency across exertion levels.

Pairwise comparison of the Exertion by Feedback Interaction

Feedback	Exertion	Exertion	Difference	StdErr	p value	Lower	Upper
**No**	38	46	0.268	0.16	0.472	-0.213	0.749
**No**	38	52	0.036	0.385	1	-1.121	1.192
**No**	38	63	0.115	0.351	0.997	-0.941	1.17
**No**	38	70	-1.194	0.341	0.018	-2.22	-0.169
**No**	46	52	-0.232	0.375	0.97	-1.36	0.896
**No**	46	63	-0.153	0.338	0.991	-1.17	0.864
**No**	46	70	-1.462	0.35	0.004	-2.513	-0.411
**No**	52	63	0.079	0.397	1	-1.115	1.273
**No**	52	70	-1.23	0.334	0.012	-2.233	-0.226
**No**	63	70	-1.309	0.3	0.003	-2.212	-0.406
**yes**	38	46	0.056	0.142	0.994	-0.372	0.484
**yes**	38	52	-0.57	0.3	0.35	-1.471	0.332
**yes**	38	63	-1.7	0.377	0.002	-2.836	-0.565
**yes**	38	70	-2.273	0.535	0.004	-3.883	-0.663
**yes**	46	52	-0.626	0.217	0.065	-1.279	0.028
**yes**	46	63	-1.756	0.375	0.001	-2.886	-0.627
**yes**	46	70	-2.329	0.451	<0.001	-3.685	-0.973
**yes**	52	63	-1.131	0.355	0.035	-2.198	-0.063
**yes**	52	70	-1.703	0.302	<0.001	-2.611	-0.795
**yes**	63	70	-0.573	0.477	0.752	-2.008	0.863

SM Table 11. Pairwise comparisons of exertion levels (38% - 70% MVC) on maintenance consistency, separated by feedback condition. The table presents the mean differences

between exertion levels, standard errors (StdErr), p-values, and 95% confidence intervals (lower and upper bounds). Significant differences ($p < 0.05$) indicate systematic changes in consistency across exertion levels.

1.6 Experiment 2 - Initial force estimation

Pairwise comparison of the Exertion Main Effect

Exertion	Exertion	Difference	StdErr	p value	Lower	Upper
**35**	50	3.05	0.519	<0.001	1.731	4.369
**35**	65	6.73	1.49	0.001	2.946	10.514
**50**	65	3.68	1.109	0.01	0.863	6.497

SM Table 12. Pairwise comparisons of exertion levels (35% - 65% MVC) on initial force estimation. The table presents the mean differences between exertion levels, standard errors (StdErr), p-values, and 95% confidence intervals (lower and upper bounds). Significant differences ($p < 0.05$) indicate systematic changes in force estimation across exertion levels.

Pairwise comparison of the Feedback Main Effect

Feedback	Feedback	Difference	StdErr	p value	Lower	Upper
**Early**	Late	4.285	1.045	0.003	1.348	7.223
**Early**	No	4.554	0.891	<0.001	2.05	7.059
**Early**	Yes	-0.019	0.106	0.998	-0.318	0.28
**Late**	No	0.269	0.445	0.929	-0.983	1.522
**Late**	Yes	-4.304	1.075	0.004	-7.328	-1.28
**No**	Yes	-4.573	0.901	<0.001	-7.107	-2.04

SM Table 13. Pairwise comparisons of feedback conditions on initial force estimation. The table presents the mean differences between conditions (Early, Late, No Feedback, and Yes Feedback), along with standard errors (StdErr), p-values, and 95% confidence intervals (lower and upper bounds). Significant differences ($p < 0.05$) suggest that the presence of feedback influences initial force estimation.

Pairwise comparison of the Exertion by Feedback Interaction

Feedback	Exertion	Exertion	Difference	StdErr	P value	Lower	Upper
**Early**	35	50	1.422	0.423	0.009	0.348	2.495
**Early**	35	65	5.276	1.425	0.004	1.657	8.896
**Early**	50	65	3.855	1.093	0.006	1.077	6.633
**Late**	35	50	4.352	0.641	<0.001	2.725	5.98

**Late**	35	65	8.526	1.716	<0.001	4.168	12.885
**Late**	50	65	4.174	1.334	0.015	0.786	7.563
**No**	35	50	4.852	0.694	<0.001	3.09	6.615
**No**	35	65	8.208	1.765	<0.001	3.724	12.691
**No**	50	65	3.355	1.497	0.09	-0.448	7.159
**Yes**	35	50	1.574	0.565	0.03	0.137	3.01
**Yes**	35	65	4.91	1.329	0.004	1.533	8.288
**Yes**	50	65	3.336	0.831	0.002	1.224	5.449

SM Table 14. Pairwise comparisons of exertion levels across feedback conditions. The table presents the mean differences in exertion levels (35, 50, and 65) within each feedback condition (Early, Late, No, Yes), along with standard errors (StdErr), p-values, and 95% confidence intervals (Lower, Upper). Significant differences ($p < 0.05$) indicate that feedback timing or presence influenced exertion levels.

Pairwise comparison of the Incentive by Feedback Interaction

Incentive	Feedback_1	Feedback_2	Difference	StdErr	p value	Lower	Upper
**Low**	Early	Late	4.338	0.962	0.001	1.632	7.044
**Low**	Early	No	5.356	1.034	<0.001	2.447	8.264
**Low**	Early	Yes	0.379	0.283	0.55	-0.416	1.173
**Low**	Late	No	1.018	0.465	0.162	-0.289	2.324
**Low**	Late	Yes	-3.96	0.957	0.003	-6.651	-1.268
**Low**	No	Yes	-4.977	1.003	<0.001	-7.797	-2.158
**high**	Early	Late	4.232	1.194	0.011	0.874	7.59
**high**	Early	No	3.753	0.826	0.001	1.429	6.077
**high**	Early	Yes	-0.416	0.284	0.478	-1.215	0.383
**high**	Late	No	-0.479	0.652	0.882	-2.311	1.353
**high**	Late	Yes	-4.648	1.241	0.007	-8.136	-1.16
**high**	No	Yes	-4.169	0.871	0.001	-6.618	-1.72

SM Table 15. Pairwise comparisons of feedback conditions across incentive levels. The table presents the mean differences in feedback conditions (Early, Late, No, Yes) within each incentive level (Low, High), along with standard errors (StdErr), p-values, and 95% confidence intervals (Lower, Upper). Significant differences ($p < 0.05$) indicate that incentive levels may modulate the effects of feedback on exertion.

Pairwise comparison of the Exertion by Incentive Interaction

Reward	Exertion	Exertion	Difference	StdErr	p value	Lower	Upper
**Low**	35	50	3.252	0.572	<0.001	1.798	4.705
**Low**	35	65	7.446	1.545	<0.001	3.521	11.371
**Low**	50	65	4.194	1.139	0.004	1.299	7.088
**high**	35	50	2.848	0.507	<0.001	1.56	4.136

**high**	35	65	6.015	1.475	0.002	2.268	9.761
**high**	50	65	3.166	1.126	0.029	0.306	6.027

SM Table 16. Pairwise comparisons of exertion levels within each reward condition. The table displays mean differences in exertion levels (35, 50, 65) for both low and high reward conditions, along with standard errors (StdErr), p-values, and 95% confidence intervals (Lower, Upper). Significant differences ($p < 0.05$) suggest that reward magnitude influences exertion differences across conditions.

1.7 Experiment 2 - Consistency Across Trials

Pairwise comparison of the Exertion main effect

Exertion	Exertion	Difference	StdErr	p value	Lower	Upper
**35**	50	-1.436	0.277	<0.001	-2.14	-0.732
**35**	65	-4.63	0.498	<0.001	-5.896	-3.365
**50**	65	-3.194	0.309	<0.001	-3.98	-2.409

SM Table 17. Comparison of mean differences in exertion levels with corresponding standard errors, p-values, and 95% confidence intervals. All comparisons are statistically significant ($p < 0.05$).

Pairwise comparison of the Feedback main effect

Feedback_1	Feedback_2	Difference	StdErr	p value	Lower	Upper
**Early**	Late	-4.783	0.495	<0.001	-6.176	-3.39
**Early**	No	-5	0.495	<0.001	-6.392	-3.607
**Early**	Yes	0.255	0.274	0.788	-0.515	1.025
**Late**	No	-0.217	0.186	0.656	-0.741	0.307
**Late**	Yes	5.038	0.574	<0.001	3.425	6.652
**No**	Yes	5.255	0.594	<0.001	3.585	6.926

SM Table 18. Comparison of mean differences in feedback conditions with corresponding standard errors, p-values, and 95% confidence intervals.

1.8 Experiment 2 - Sustained Force Maintenance

Pairwise comparison of the Force Main Effect

Exertion	Exertion	Difference	StdErr	P value	Lower	Upper

**35**	50	3.785	0.475	<0.001	2.577	4.992
**35**	65	7.564	1.25	<0.001	4.389	10.738
**50**	65	3.779	0.886	0.001	1.528	6.03

SM Table 19. Pairwise comparisons of exertion levels. The table presents the mean differences in exertion levels (35, 50, 65), along with standard errors (StdErr), p-values, and 95% confidence intervals (Lower, Upper). Significant differences ($p < 0.05$) indicate systematic changes in exertion across conditions.

Pairwise comparison of the Feedback Main Effect

Feedback_1	Feedback_2	Difference	StdErr	p value	Lower	Upper
**Early**	Late	-0.906	0.882	0.736	-3.388	1.575
**Early**	No	2.169	0.676	0.022	0.268	4.069
**Early**	Yes	-5.303	0.461	<0.001	-6.599	-4.008
**Late**	No	3.075	0.586	<0.001	1.428	4.722
**Late**	Yes	-4.397	1.067	0.003	-7.398	-1.396
**No**	Yes	-7.472	0.905	<0.001	-10.015	-4.928

SM Table 20. Pairwise comparisons of feedback conditions. The table presents the mean differences between feedback conditions (Early, Late, No, Total), along with standard errors (StdErr), p-values, and 95% confidence intervals (Lower, Upper). Significant differences ($p < 0.05$) indicate systematic effects of feedback on the outcome measure.

Pairwise comparison of the Force by Feedback interaction

Feedback	Exertion	Exertion	Difference	StdErr	p value	Lower	Upper
**Early**	35	50	3.71	0.483	<0.001	2.484	4.936
**Early**	35	65	8.679	1.102	<0.001	5.878	11.48
**Early**	50	65	4.969	0.752	<0.001	3.058	6.88
**Late**	35	50	4.161	0.57	<0.001	2.713	5.608
**Late**	35	65	8.003	1.559	<0.001	4.042	11.964
**Late**	50	65	3.843	1.229	0.015	0.719	6.966
**No**	35	50	6.02	0.614	<0.001	4.461	7.58
**No**	35	65	9.133	1.398	<0.001	5.581	12.685
**No**	50	65	3.113	1.066	0.023	0.406	5.82
**Yes**	35	50	1.247	0.518	0.065	-0.07	2.564
**Yes**	35	65	4.439	1.264	0.006	1.229	7.649
**Yes**	50	65	3.192	0.79	0.002	1.185	5.199

SM Table 21. Pairwise comparisons of exertion levels within feedback conditions. The table displays mean differences in exertion levels (35, 50, 65) for each feedback condition (Early, Late, No, Yes), along with standard errors (StdErr), p-values, and 95% confidence intervals (Lower, Upper). Significant differences ($p < 0.05$) suggest that feedback influenced exertion level comparisons.

Pairwise comparison of the Force by Incentive Interaction

Incentive	Exertion	Exertion	Difference	StdErr	p value	Lower	Upper
**Low**	35	50	4.099	0.576	<0.001	2.636	5.561
**Low**	35	65	8.333	1.359	<0.001	4.881	11.784
**Low**	50	65	4.234	0.928	0.001	1.876	6.592
**high**	35	50	3.47	0.401	<0.001	2.453	4.488
**high**	35	65	6.795	1.181	<0.001	3.795	9.794
**high**	50	65	3.324	0.899	0.004	1.041	5.607

SM Table 22. Pairwise comparisons of exertion levels within incentive conditions. The table presents mean differences in exertion levels (35, 50, 65) for each incentive condition (Low, High), along with standard errors (StdErr), p-values, and 95% confidence intervals (Lower, Upper). Significant differences ($p < 0.05$) indicate that incentives influenced exertion level comparisons.

1.9 Experiment 2 - Deviation within trials in Force Maintenance

Pairwise comparison of the Force Main effect

Exertion	Exertion	Difference	StdErr	p value	Lower	Upper
**35**	50	-0.458	0.076	<0.001	-0.65	-0.266
**35**	65	-1.23	0.116	<0.001	-1.525	-0.936
**50**	65	-0.772	0.074	<0.001	-0.961	-0.583

SM Table 23. Pairwise comparisons of exertion levels. The table presents mean differences in exertion levels (35, 50, 65), along with standard errors (StdErr), p-values, and 95% confidence intervals (Lower, Upper). All comparisons are statistically significant ($p < 0.05$), indicating a systematic decrease in exertion across levels.

Pairwise comparison of the Feedback Main Effect

Feedback_1	Feedback_2	Difference	StdErr	p value	Lower	Upper
**Early**	Late	0.767	0.135	<0.001	0.387	1.147
**Early**	No	-0.073	0.09	0.85	-0.327	0.181
**Early**	Yes	0.422	0.157	0.064	-0.02	0.863
**Late**	No	-0.84	0.106	<0.001	-1.137	-0.542
**Late**	Yes	-0.345	0.162	0.178	-0.799	0.109
**No**	Yes	0.494	0.175	0.049	0.001	0.987

SM Table 24. Pairwise comparisons of feedback conditions. The table presents mean differences between feedback conditions (Early, Late, No, Total), along with standard errors (StdErr), p-values, and 95% confidence intervals (Lower, Upper). Significant differences ($p < 0.05$) were observed between Late and No feedback, as well as between No and Total feedback, indicating meaningful variations in responses across feedback types.

Pairwise comparison of the Force by Feedback Interaction

Feedback	Exertion	Exertion	Difference	StdErr	p value	Lower	Upper
**Early**	35	50	-0.45	0.159	0.028	-0.855	-0.046
**Early**	35	65	-1.487	0.202	<0.001	-2.001	-0.973
**Early**	50	65	-1.037	0.198	<0.001	-1.54	-0.534
**Late**	35	50	-0.202	0.046	0.001	-0.319	-0.085
**Late**	35	65	-0.677	0.105	<0.001	-0.943	-0.411
**Late**	50	65	-0.475	0.102	<0.001	-0.734	-0.217
**No**	35	50	-0.554	0.104	<0.001	-0.817	-0.291
**No**	35	65	-1.128	0.154	<0.001	-1.52	-0.736
**No**	50	65	-0.574	0.134	0.001	-0.915	-0.232
**Yes**	35	50	-0.626	0.175	0.005	-1.069	-0.182
**Yes**	35	65	-1.629	0.234	<0.001	-2.224	-1.035
**Yes**	50	65	-1.004	0.165	<0.001	-1.423	-0.584

SM Table 25. Pairwise comparisons of exertion levels across feedback conditions. The table presents the mean differences in exertion levels (35, 50, 65) within each feedback condition (Early, Late, No, Total), along with standard errors (StdErr), p-values, and 95% confidence intervals (Lower, Upper). Significant differences ($p < 0.05$) were observed across all comparisons, indicating a consistent decline in exertion levels across feedback conditions.

1.10 Experiment 2 - Consistency within trials in Force Maintenance

Pairwise comparison of the Force Main Effect

Exertion	Exertion	Difference	StdErr	p value	Lower	Upper
**35**	50	-0.641	0.098	<0.001	-0.889	-0.392
**35**	65	-2.414	0.266	<0.001	-3.089	-1.738
**50**	65	-1.773	0.196	<0.001	-2.272	-1.274

SM Table 26. Pairwise comparisons of exertion levels. The table presents the mean differences in exertion levels (35, 50, 65), along with standard errors (StdErr), p-values, and 95% confidence intervals (Lower, Upper). Significant differences ($p < 0.05$) were observed across all comparisons, indicating a progressive decrease in exertion measures as intensity increased.

Pairwise comparison of the Feedback Main Effect

Feedback_1	Feedback_2	Difference	StdErr	p value	Lower	Upper
**Early**	Late	-1.906	0.188	<0.001	-2.434	-1.379
**Early**	No	-1.364	0.209	<0.001	-1.953	-0.776

**Early**	Yes	1.133	0.233	0.001	0.477	1.788
**Late**	No	0.542	0.163	0.017	0.084	1.001
**Late**	Yes	3.039	0.325	<0.001	2.125	3.953
**No**	Yes	2.497	0.335	<0.001	1.554	3.44

SM Table 27. Pairwise comparisons of feedback conditions. The table presents mean differences between feedback conditions (Early, Late, No, Total), along with standard errors (StdErr), p-values, and 95% confidence intervals (Lower, Upper). Significant differences ($p < 0.05$) were observed between Late and No feedback, as well as between No and Total feedback, indicating meaningful variations in responses across feedback types.

How visual and proprioceptive feedback mediate the effect of monetary incentive on motor precision

Menghi N.[1#], Coricelli, G.[2,3], Hickey, C.[4]

1 Max Planck for Human Cognitive and Brain Sciences;
Department of Psychology, Leipzig, Germany
2 Department of Economics
University of Southern California, Los Angeles, USA
3 Laboratory for the Psychology of Child Development (LaPsyDÉ), UMR CNRS 8240, Paris, France
4 Centre for Human Brain Health and School of Psychology, University of Birmingham, Birmingham, United Kingdom
# Corresponding author: menghi@cbs.mpg.de

For Review Only

Abstract

This paper investigates the relationship between motor precision, visual feedback, and monetary incentives in 2 experiments. In both, participants exerted force via a hand dynamometer to maintain force production at identified levels while we manipulated the quality of visual feedback regarding their performance. In Experiment 1, monetary incentives improved motor performance only when visual feedback was provided. In Experiment 2, we simplified target representation by reducing the number of targets, making them easier to distinguish via proprioception and somatosensation. Under these conditions, incentives enhanced performance even without visual feedback. These findings suggest that while visual feedback is key to mediating motivational effects on fine motor control, incentives can also directly enhance performance when targets are easily represented through proprioceptive cues.

Keywords: Sensory Feedback; Motor Control; Incentives

1 Introduction

Daily activities demand precise control of force generation. As a real-world example, consider a waiter balancing a tray loaded with dishes. This individual must maintain fine gradation of force to sustain tray balance while navigating through a busy, dynamic environment. This kind of force generation clearly relies on monitoring of somatosensory and proprioceptive feedback (Whittier, Patrick, & Fling, 2023). Our waiter will be acutely aware of the position of his hand and the force created by the weight of the tray. However, he will also visually monitor his performance, and this is an example of how fine motor behaviour is also guided by visual feedback (Goodale & Milner, 1992; Milner & Goodale, 2008).

In the lab, results show that raw accuracy in force generation generally decreases as required force magnitude increases, but that visual feedback mitigates this pattern and improves accuracy (Limonta, Rampichini, Cè, & Esposito, 2015; Noble, Eng, & Boyd, 2013). When visual feedback is entirely removed, force tends to diminish and drift (Mayhew, Porcaro, Tecchio, & Bagshaw, 2017; Vaillancourt, Slifkin, & Newell, 2001; Abolins & Latash, 2022; Abolins, Ormanis, & Latash, 2023). Similarly, overall variability in force generation increases as a function of required force (Vaillancourt & Russell, 2002), but reduces when visual feedback is provided, stabilizing performance (Vaillancourt, Thulborn, & Corcos, 2003; Slifkin, Vaillancourt, & Newell, 2000; Baweja, Kennedy, Vu, Vaillancourt, & Christou, 2010).

Performance is also sensitive to motivational incentive, which wields significant influence over force generation and fine motor performance (Manohar

For Review Only

et al., 2015; Adkins, Gary, & Lee, 2021). The prospect of monetary reward potentiates participant willingness to engage in an action involving force generation (Klein-Flügge, Kennerley, Friston, & Bestmann, 2016; Apps, Grima, Manohar, & Husain, 2015; Croxson, Walton, O'Reilly, Behrens, & Rushworth, 2009; Le Bouc et al., 2016) and energizes force contraction (Zénon, Devesse, & Olivier, 2016; Pessiglione et al., 2007; Oudiette, Vinckier, Bioud, & Pessiglione, 2019). It also impacts the trade-off between force exertion and rest (Meyniel, Sergent, Rigoux, Daunizeau, & Pessiglione, 2013; Müller, Klein-Flügge, Manohar, Husain, & Apps, 2021). When the restaurant is busy and there is money to be earned, our waiter will maintain his performance despite increased pace and heavier loads.

Each of these influences on force generation – the effect of visual feedback and the effect of incentive motivation – have been individually investigated at considerable depth, but their interaction has been relatively underexplored. There are a range of possibilities here. At one extreme, the effect of incentive motivation on force generation may be strongly mediated by the monitoring of visual feedback. By this, the prospect of reward may act in large part by motivating individuals to track visual feedback regarding the accuracy and efficacy of performance so this can be used to optimize behaviour. At the other extreme is the possibility that the effect of motivation on motor performance is independent of visual feedback. This could mean that motivation acts directly to accentuate motor control, or that motivation influences how individuals use somatosensory and proprioceptive information to optimize their behaviour. When our waiter is motivated by monetary prospect to work harder, does this reflect increased consideration of the visual position and tilt of his tray? Or does he more carefully monitor

For Review Only

proprioceptive information about his hand position and force exertion? If both, how much does his ability to improve performance rely on visual feedback on task performance?

We conducted 2 experiments to investigate this issue. Our general experimental paradigm draws inspiration from previous research investigating motor control and the impact of incentives on maximal force exertion (eg. Pessiglione et al., 2007). Participants were asked to exert force via a hand dynamometer to target levels that were defined as a percentage of maximum voluntary contraction. They were informed at the beginning of each trial that a cash reward could be earned for accurate task performance, and we manipulated the magnitude of this reward across trials (20¢ vs 1¢). We also independently manipulated the availability of visual feedback on performance accuracy. In some trials participants were provided continuous, online feedback about how closely their performance approached the target level of force generation. In other trials, this information was limited to the initial estimation of force generation, or to the later maintenance of force, or was absent altogether. Our aim was to assess how the impact of incentive motivation on force generation was influenced by change in the presence and quality of visual feedback.

To foreshadow, in Experiment 1 we find that when visual feedback is removed from our task, participants show no motivational benefit to task performance. In the confines of this experiment, the impact of motivation on fine force control appears entirely mediated by the visual feedback on performance accuracy. However, in Experiment 1 we employ a large range of target forces, and this may have made it difficult for participants to represent these targets in terms of proprioception and somatosensation. Experiment 2 was designed to determine if

For Review Only

motivation would impact performance when there were fewer potential force targets, such that these might be better distinguished in terms of proprioception. This led to re-emergence of motivation effects when visual feedback was absent or limited in duration. Our results show that visual feedback plays a key mediating role in the effect of motivation on force generation, in particular when target performance is subtle and difficult to represent via proprioception alone.

2 Methods

2.1 Participants

To ensure a final sample size of 20 participants per experiment, we initially recruited twenty-two participants (12 females, 10 males; mean age 24.3 years; range 20-30 years) for experiment 1 and a separate group of 22 participants (12 females, 10 males; mean age 24.3; range 20-31) for experiment 2. The sample size of Experiment 1 was not guided by formal power analysis as we had no prediction of effect size. The sample size for Experiment 2 was selected under the assumption that key effect sizes would be similar across experiments.

Two male participants were excluded from the analysis of experiment 1 and 2 participants, 1 male and 1 female, were excluded from the analysis of experiment 2. Three of these excluded participants commonly failed to respond, particularly in experimental conditions where earnings were reduced, resulting in force error and force variance that was more than 3 standard deviations from the group mean. The fourth participant consistently exerted force that was substantially over the target, suggesting inaccuracy in the calibration of maximum force that preceded experimental participation. The participants were all right-handed and naive to the purpose of the experiment. Participants were paid based on performance, with pay varying between 5 and 15 euros in experiment 1 and between 10 and 21 euros in

For Review Only

experiment 2. All gave informed written consent and the study procedure was approved by the local institutional review board of the University of Trento.

2.2 Apparatus and Stimuli

In both experiments, participants sat at approximately 60 cm from a computer monitor (VIEWPixx/EEG 22”; 1920x1080; 120 Hz) in a dimly illuminated room with their right hand laying over the table grasping a hand dynamometer. The dynamometer (HD-BTA Vernier) was used to record power grip force effort in Newtons (N) with an accuracy of ±0.6 N. This dynamometer is a strain-gauge-based isometric force sensor which amplifies force and converts it into a voltage signal. The voltage signal was transferred to an Arduino Uno through Vernier interface shield hardware and subsequently to an acquisition computer. The force exerted by participants in experiment 1 ranged from 133 to 350 N, while in experiment 2, it ranged from 139 to 394 N. These values remained well within the sensor's operational range of 0–600 N, ensuring accurate measurements. The force signal was sampled at 50 Hz in experiment 1 and at 80 Hz in experiment 2. During the experiments, signals from this sensor were sent to MATLAB (The Mathworks Inc.) for visual real-time feedback of participant’s effort exertion. Feedback was updated at a frequency rate of 25 Hz in experiment 1 and 20 Hz in experiment 2. Presentation of visual stimuli and acquisition of behavioural data was accomplished using PsychToolBox (Brainard, 1997) and custom MATLAB scripts. Before beginning each experiment participants were requested to exert the most force they could on the dynamometer 3 times, each time for 3 s., with 10 s. of rest between each instance. The maximal voluntary contraction (MVC) was computed as the average of the highest peaks achieved per trial, following a simplified approach based on Slifkin and Newell (1999).

For Review Only

Experiment 1 was designed to investigate how reward incentivization interacts with visual feedback during a task requiring force exertion and maintenance. The trial sequence is illustrated in Fig. 1A. Each experimental trial began with a cue indicating the incentive condition (20 cents or 1 cent) then a target force appeared, which was randomly selected from 5 possibilities and calculated as a percentage of MVC (38%, 46%, 54%, 62%, and 70%). Participants attempted to match this target force level with the hand dynamometer using a whole-hand power grip. For half of the trials, participants were presented with online visual feedback, while for the other half, no visual feedback was provided. When visual information was present, feedback took the form of a stylized black thermometer that was displayed at the centre of an otherwise uniform dark grey background. The thermometer became increasingly red as force was exerted on the dynamometer and a green square on the thermometer indicated the target force output. When visual feedback was absent, the thermometer appeared but did not move.

Task performance lasted 3 s. and began with an auditory tone indicating the beginning of a 1 s. force estimation period, in which participants should adjust the force to the target value. A tone subsequently indicated the beginning of a 2 s. maintenance period and a final tone indicated the end of the trial. Experiment 1 took about 2 hours to complete and was composed of 15 practice trials followed by 300 experimental trials in 15 blocks, with breaks between blocks.

In each trial, participants received a percentage of the incentive value cued at the beginning of the trial, with the specific percentage determined by the quality of task performance. This gain was determined based on the deviation of the exerted force from the target force using the following procedure: The error was calculated

For Review Only

as the square root of the average quadratic difference between the participant's exertion at each time point t during the maintenance period and the target force:

$$\text{Error} = \sqrt{\frac{\sum_t^T (\text{trial\_exertion}(t) - \text{target})^2}{T}}$$

The participant's gain was then calculated by multiplying the incentive condition (high: 20 cents or low: 1 cent) by the proportion of the subject-specific range (calculated as 4% of their maximum exertion) adjusted by the error:

$$\text{Gain} = \frac{\text{Incentive} * \text{dev}}{\text{Error}}$$

This method accounts for the participant's performance in relation to their maximum exertion and the error from the target force, ensuring that the reward reflects both accuracy and effort. Participants were instructed that both overshoot and undershoot were penalized and were explicitly aware of the relationship between their performance and their pay.

As described below, results suggested that participants may have had difficulty representing or reproducing the large number of subtly differing force target values that were employed in Experiment 1. To test this, Experiment 2 employed only three force target values (35%, 50% and 65% of MVC). Experiment 2 additionally included two new feedback conditions designed to investigate the role of feedback in the control versus maintenance of force exertion. The total feedback (TF) and no feedback (NF) conditions described above were joined by early feedback (EF) and late feedback (LF) conditions. In the EF condition, force feedback was provided only for the first 1.5 seconds of task performance, then disappeared with the onset of the second tone. In the LF condition force feedback was provided 1.5 seconds after the beginning of performance and sustained for 2.5 s. until the end of the trial. As in Experiment 1, task performance began with an

For Review Only

auditory tone indicating the need for force estimation, followed 1.5 s. later by a tone indicating the beginning of a 2.5 s. maintenance period before a final tone indicated the end of the trial.

Importantly, feedback in the LF condition was not a direct reflection of actual force, but rather reflected variance in performance from a normalized baseline established at the beginning of the feedback period. That is, the force recorded at the start of feedback was set in the visual feedback as equivalent to the current target force. This meant that force feedback always began at the target level, with subsequent deviation reflecting variance from the force magnitude established at the beginning of the feedback interval. This approach was adopted in order to provide participants with an accurate reflection of variance in their performance during the maintenance period without providing information regarding absolute accuracy.

As in Experiment 1, there were two incentive conditions in Experiment 2 (1 cent and 20 cents) that were cued at the beginning of each trial. An additional, concurrent cue indicated the type of feedback in the trial, such that participants could prepare for the offset of feedback (in the EF condition) or the onset of feedback (in the LF condition). As illustrated in Figure 1B, an empty square indicated a NF trial; a fully black square indicated a TF trial; a square with the left side black indicated an EF trial; a square with the right side black indicated a LF trial. All conditions were randomized and counterbalanced across trials and the experiment was composed of 24 practice trials followed by 360 experimental trials divided into 15 blocks.

For Review Only

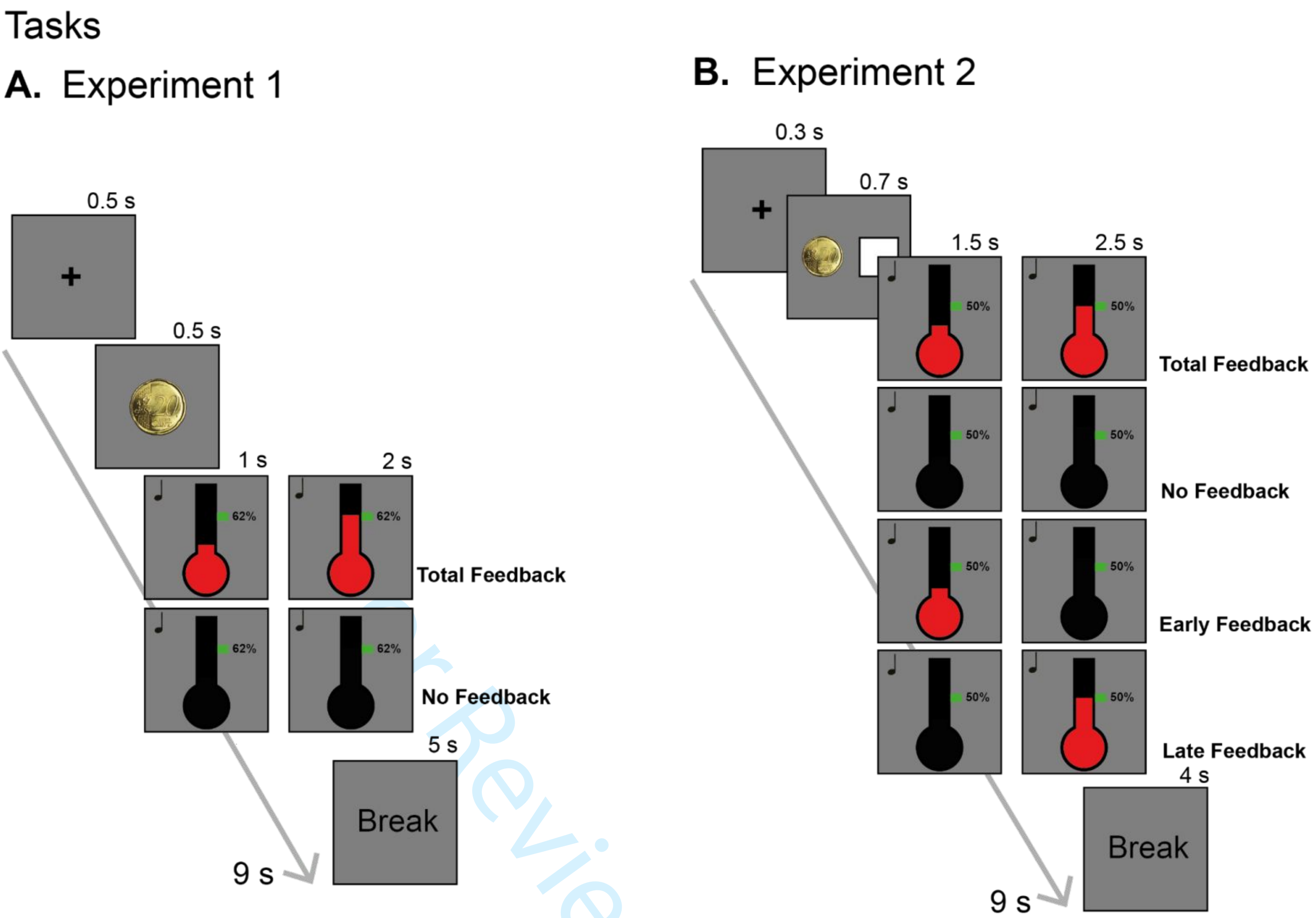

Figure 1: Task schematics. A Experiment 1. Each trial started with the presentation of a fixation cross (500 ms), followed by an image of the incentive that could be won in the trial (500 ms). An auditory stimulus subsequently identified the beginning of the trial and the feedback display appeared. The feedback, if present, was displayed as a red fluid in a stylized thermometer shape. The task lasted 3 seconds, 1 second of force estimation and 2 seconds of maintenance (both signalled by an auditory stimulus), followed by an invitation to relax the hand for 5 seconds. Each trial lasted 9 seconds in total. Participants received feedback during both force estimation and maintenance (Total feedback condition) or no feedback throughout the task (No Feedback). At the end of each block, participants were shown a message to relax and given information about the cumulative reward earned during that block. B Experiment 2. Each trial started with the presentation of a fixation cross for 300 ms, followed by the presentation of two cues (700 ms) that provided information about both feedback and incentive conditions. An auditory stimulus subsequently identified the beginning of the trial and the visual feedback, if present, appeared. Feedback was provided as in Experiment 1. The task lasted 4 seconds, 1.5 seconds of force estimation and 2.5 seconds of maintenance (both signalled by an auditory stimulus), followed by an invite to relax the hand for 4 seconds, for a total of 8 seconds per trial. In this experiment, two new feedback conditions were introduced: Early Feedback, in which feedback was present during force estimation only, and Late Feedback, in which feedback was present during force maintenance only. As in Experiment 1, information about the cumulative sum of reward earned during the block was provided at the end of the block.

3 Experiment 1 - Data Analysis

Our main goal was to determine if incentives affect accuracy in force estimation and maintenance, and if this interacts with the availability of visual feedback information. We divide the analysis into two parts. First, we characterize force estimation as the average signed error from the target during 10 data-points (0.4 s) after the end of the estimation period (See Fig. 2). We also calculate the consistency of this signal across trials, which is defined as the standard deviation of the mean estimation across trials within a participant. Second, we characterize force maintenance as the averaged error from the target during the maintenance period (See Fig. 2), additionally calculating variability in this signal (defined as the deviation within a trial), and the consistency of this signal across trials (defined as the standard deviation of the mean maintenance across trials).

To analyze the data, we employ repeated-measures ANOVAs to assess the effects of exertion targets, incentives and visual feedback on force estimation and maintenance. Further post hoc comparisons can be found in the supplementary materials. We define statistical significance at $p < 0.05$ and consider results with $0.05 \le p < 0.1$ as trends. Uncorrected results are reported in the paper, but all interpreted statistical tests remain reliable if Greenhouse-Geisser correction for violations of sphericity is adopted.

For Review Only

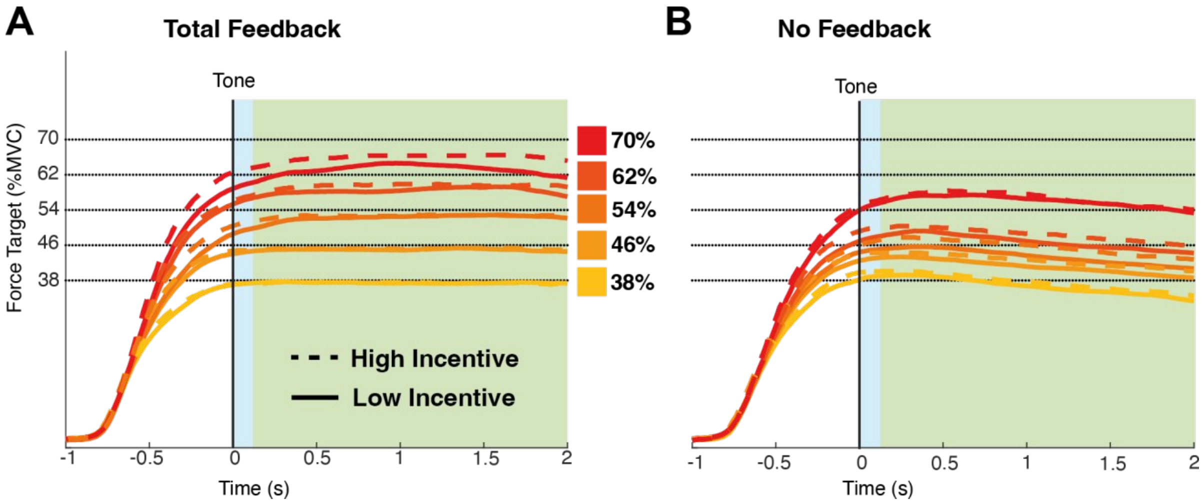

Figure 2: Force estimation and maintenance in Experiment 1 Panels A-B show the average performance across participants in the total feedback and no feedback conditions. The interval highlighted in blue was defined as force estimation. The interval highlighted in green was defined as force maintenance.

For Review Only

4 Results

4.1 Initial force estimation

Initial force estimation was computed as the average distance from the target of the 10 data points after the presentation of the auditory tone that indicated the end of the estimation period (Fig. 2). Force estimation was analyzed in a three-way mixed model analysis of variance (ANOVA) with factors for exertion (5 levels: 38 - 70% MVC), incentive (2 levels: 1 cent vs. 20 cents), and feedback (2 levels: total feedback vs. no feedback). This identified significant main effects of exertion ($F_{4,76} = 55.525$, $p < 0.001$) and feedback ($F_{1,19} = 13.999$, $p = 0.001$), alongside a trend toward a main effect of incentive ($F_{1,19} = 4.162$, $p = 0.055$). Participants performed better in the total feedback condition (average error ~3%) than in the no feedback condition (average error ~6%) and in lower exertion conditions (see supplementary materials for post hoc analysis; SM Table 1 and 2). A significant interaction of feedback and exertion emerged ($F_{4,76} = 22.568$, $p < 0.001$)

alongside a critical 3-way interaction ($F_{4,76} = 2.829$, $p = 0.030$). The 3-way interaction was driven by a general increase in the effect of incentive with greater exertion requested, but only in the feedback condition (see supplementary materials section 1.1 for post hoc analysis). No other effects reached significance (exertion * incentive: $F_{4,76} = 0.929$, $p = 0.452$; feedback * incentive: $F_{1,19} = 0.183$, $p = 0.673$).

These results are illustrated in Figure 3. To summarize, participants undershot the target and this tended to increase as exertion requested increased. Performance was improved by feedback and by high incentives (See Fig. 3A-B). The effect of incentive was most pronounced in difficult trials when feedback was available (Fig. 3C), with this pattern absent when feedback was absent (Fig. 3D).

For Review Only

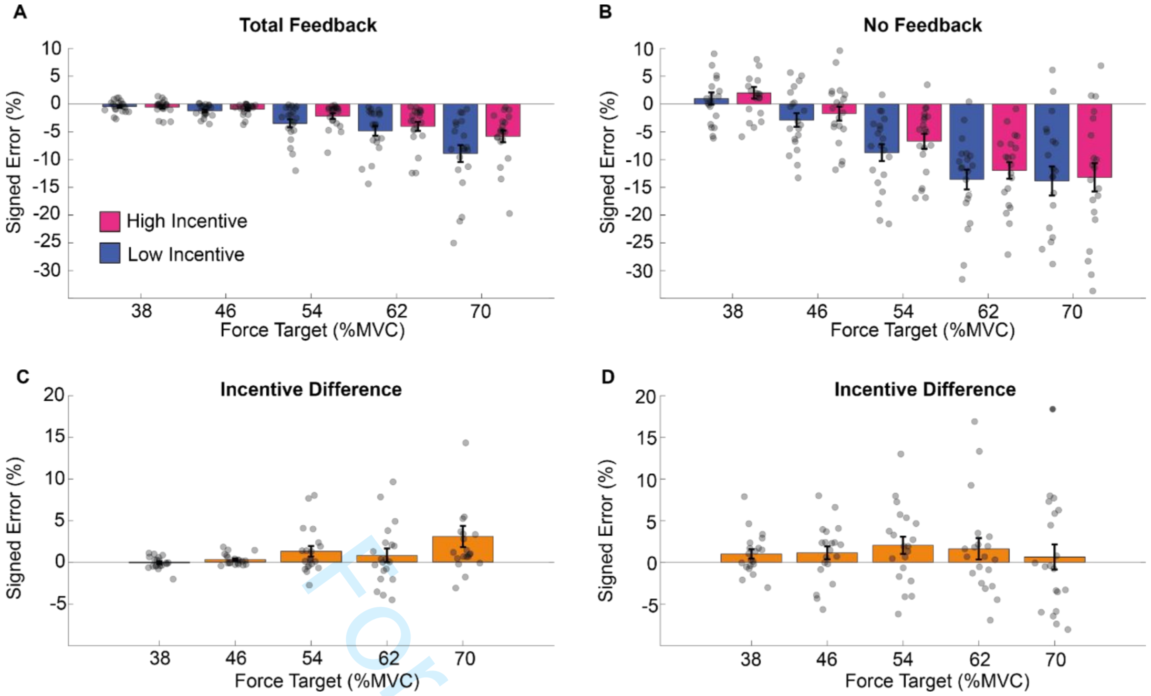

Figure 3: Mean Force Estimation in Experiment 1 Panels A-B show the signed error from the target, during the force estimation period for the total feedback and the no feedback condition. Panels C-D show the difference between high and low incentive conditions for each target force level in the total feedback condition. In this and subsequent figures, each dot represents mean performance for a single participant and error bars represent standard error of the mean.

4.1.1 Consistency across trials

Consistency was computed as the standard deviation of the mean force estimation over trials within a participant. Consistency was analyzed in a three-way model analysis of variance (ANOVA) with factors for exertion (5 levels: 38 - 70% MVC), incentive (2 levels: 1 cent vs. 20 cents), and feedback (2 levels: total feedback vs. no feedback). This identified significant main effects for exertion ($F_{4,76} = 31.748$, $p < 0.001$) and feedback ($F_{1,19} = 82.572$, $p = 0.001$), alongside a trend toward an effect of incentive ($F_{1,19} = 4.137$, $p = 0.056$). A significant interaction of exertion and feedback also emerged ($F_{4,76} = 2.756$, $p = 0.033$), as did an interaction of exertion and incentive ($F_{4,76} = 3.118$, $p = 0.019$).

For Review Only

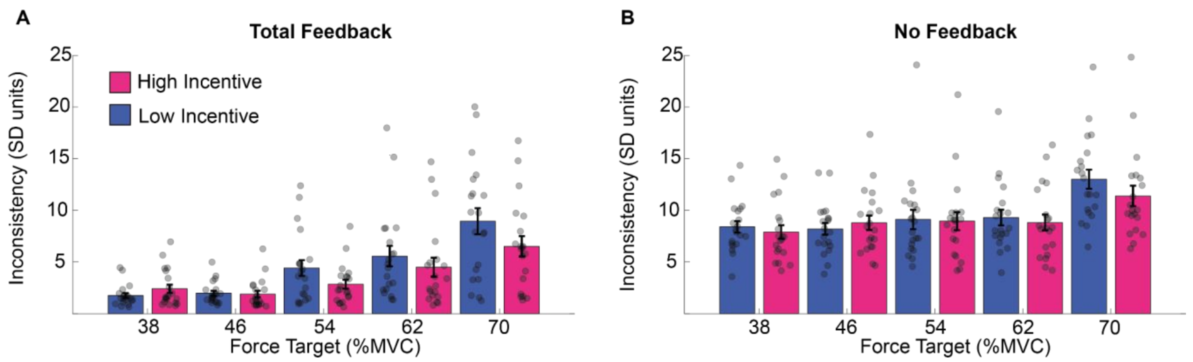

Figure 4: Consistency of force estimation across trials in Experiment 1 Panels A-B. Performance consistency in estimating the force requested across trials for the total feedback condition and the no feedback condition. Consistency is represented in standard deviation units, thus smaller values reflect increased consistency.

For Review Only

No other effects reached significance (feedback*incentive: $F_{1,19} = 1.911$, $p = 0.182$; exertion*feedback*incentive: $F_{4,76} = 1.014$, $p = 0.405$) (see supplementary materials for posthoc analysis of the direction of the effect SM Tables 3 and 4).

These results are illustrated in Figure 4. Force estimation was more consistent when feedback was present, and consistency decreased as exertion increased. High incentives increased participants' consistency, especially when requested exertion was high, but this pattern was not reliably sensitive to the manipulation of feedback.

4.2 Sustained Force Maintenance

Sustained force maintenance was computed as the average distance from the target of the data points after the end of the estimation period until the end of the trial (See Fig. 2). Sustained force was analyzed in a three-way mixed model analysis of variance (ANOVA) with factors for exertion (5 levels: 38 - 70% MVC), incentive (2 levels: 1 cent vs. 20 cents), and feedback (2 levels: total feedback vs. no feedback). This identified all three main effects (exertion: $F_{4,76} = 43.876$, $p < 0.001$; feedback: $F_{1,19} = 46.662$, $p < 0.001$; incentive: $F_{1,19} = 4.579$, $p = 0.0455$). Participants performed better in the total feedback condition (average error ~2%)

than in the no feedback condition (average error ~9%) and for lower levels of exertion than for higher levels of exertion (see supplementary materials SM Table 5 for posthoc analysis). An interaction between exertion and feedback also emerged ($F_{4,76} = 25.59$, $p < 0.001$) as did the 3-way interaction ($F_{4,76} = 4.844$, $p = 0.001$). No other effects reached significance (exertion * incentive: $F_{4,76} = 0.8313$, $p = 0.213$; feedback * incentive: $F_{1,19} = 1.657$, $p = 0.213$) (see supplementary materials section 1.3 and SM Table 6 for post hoc analysis). These results are illustrated in Figure 5. Error increased with exertion, but was reduced by visual feedback and incentive (Fig. 5A-B). The effect of incentive was most pronounced in difficult trials when feedback was provided (Fig. 5C), but this pattern did not emerge when feedback was absent (Fig. 5D).

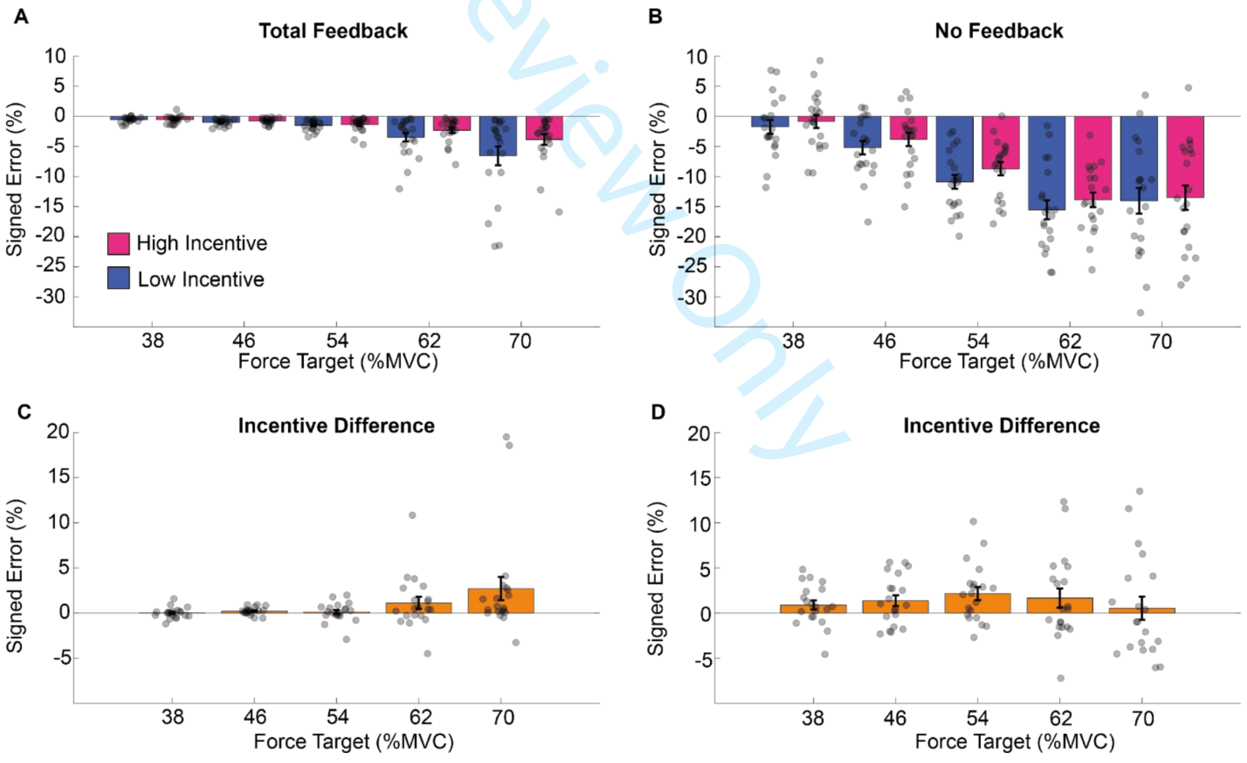

Figure 5: Mean Force maintenance in Experiment 1 Panel A-B. shows the mean of the error from the target (Y-axis) during the force maintenance period in the total feedback and during the no feedback, at each force level (X-axis) and per incentive condition. Force error was defined as the difference at each time point between the observed force level and the current target. Positive values therefore reflects performance overshoot, and negative values undershoot. Panel C and D show the difference between high and low incentive, per force and feedback conditions.

For Review Only

4.2.1 Deviation within trials

Deviation was computed as the standard deviation of the force exerted during the maintenance period. Before calculating the standard deviation, exertion data was detrended to remove the linear drift in performance over the course of the trial. A higher standard deviation represents higher variability during the exertion. Deviation was analyzed in a three-way model analysis of variance (ANOVA) with factors for exertion (5 levels: 38 - 70% MVC), incentive (2 levels: 1 cent vs. 20 cents), and feedback (2 levels: total feedback vs. no feedback). This identified significant main effects of exertion ($F_{4,76} = 23.149$, $p < 0.001$) and feedback ($F_{1,19} = 16.764$, $p < 0.001$), alongside a trend toward an effect of incentive ($F_{1,19} = 3.732$, $p = 0.068$). Participants deviated more in the no feedback condition (average standard deviation (SD) ~3%) than in the total feedback condition (average SD ~2%) and for lower rather than higher levels of exertion (see supplementary materials SM Table 7 for post hoc analysis). The interaction of feedback and exertion was significant ($F_{4,76} = 8.617$, $p < 0.001$), as was the interaction of exertion by incentive ($F_{4,76} = 2.972$, $p = 0.024$). No other effects reached significance (feedback*incentive: $F_{1,19} = 3.267$, $p = 0.865$; exertion*feedback*incentive: $F_{4,76} = 1.768$, $p = 0.143$) (see supplementary materials SM Table 8 and 9 for post hoc analysis).

These results are illustrated in Figure 6. Deviation increased with exertion requested, but was reduced by visual feedback and incentives (Fig. 6). The impact of incentive was greatest when the task was most difficult. While this effect of incentive appears larger in the feedback condition, this was not statistically

For Review Only

significant.

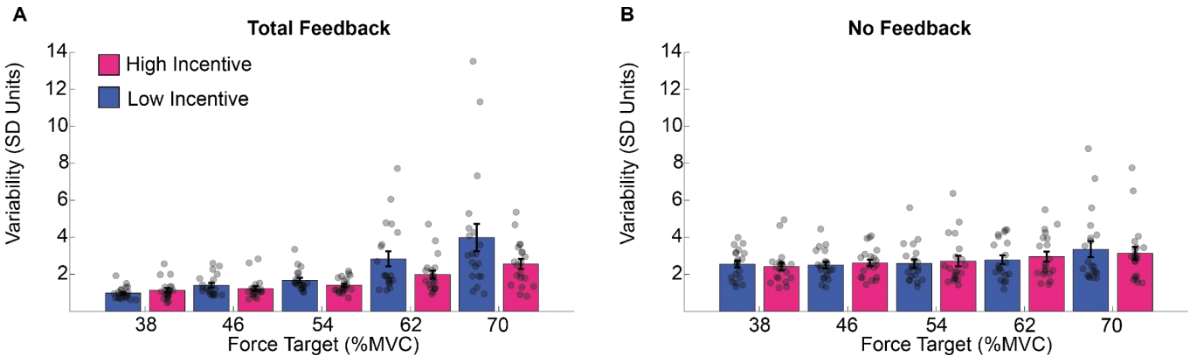

Figure 6: Deviation from the target in Experiment 1 Panel A-B show the averaged standard deviation (SD) within trials for the total feedback condition , and the no feedback condition , per force (x-axis) and incentive condition. We averaged the standard error of force across time points in the force maintenance period (y-axis).

4.2.2 Consistency across trials

Consistency was computed as the standard deviation of the mean force exertion over trials within each participant. This represents a measure of the reliability of participants' average maintenance. A higher standard deviation represents low consistency over trials. Consistency was analyzed in a three-way model analysis of variance (ANOVA) with factors exertion (5 levels: 38 - 70% MVC), incentive (2 levels: 1 cent vs. 20 cents), and feedback (2 levels: total feedback vs. no feedback). This identified main effects of exertion ($F_{4,76} = 16.707$, $p < 0.001$) and feedback ($F_{1,19} = 111.66$, $p = 0.001$). Participants were less consistent in the no feedback condition (Standard Deviation of the average ~4.5%) compared to the total feedback condition (~1.5%) and more consistent for lower rather than higher levels of exertion (see supplementary materials SM Table 10 for post hoc analysis).

The exertion by feedback interaction was also significant ($F_{4,76} = 4.718$, $p = 0.001$). No other effects reached significance (incentive: $F_{1,19} = 0.397$, $p = 0.535$; exertion*incentive: $F_{4,76} = 1.51$, $p = 0.207$; feedback*incentive: $F_{1,19} = 1.001$, $p = 0.329$; exertion*feedback*incentive: $F_{4,76} = 1.196$, $p = 0.319$) (see supplementary materials SM Table 11 for post hoc analysis).

For Review Only

These results are illustrated in Figure 7. Force maintenance became less consistent as exertion increased, and this was acute in the feedback condition. Incentive had no reliable impact on any pattern in this data.

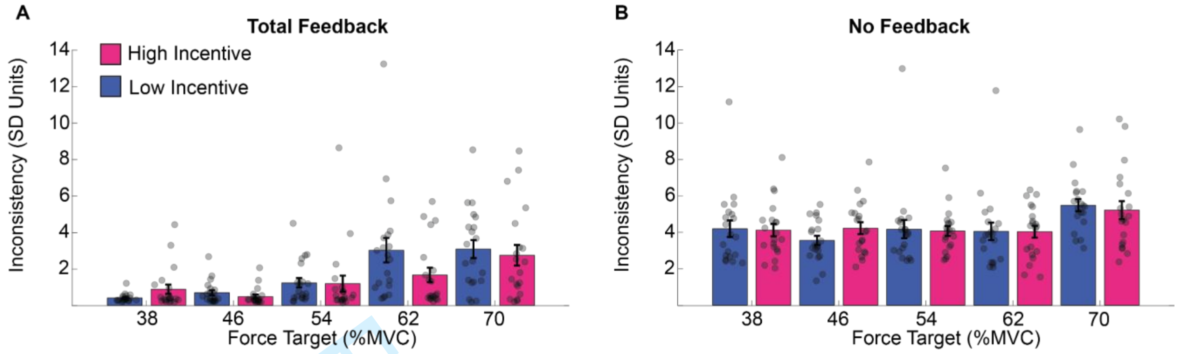

Figure 7: Consistency of force estimation in Experiment 1 Panel A-B shows the results of the standard deviation of the mean exertion across trials (y-axis) during the total feedback condition and the no feedback condition, per force (x-axis) and incentive condition.

4.3 Summary of results from Experiment 1

These results suggest that visual feedback is necessary in order for incentive motivation to impact force generation. However, the task we employed in Experiment 1 involved five exertion levels, and one possibility is that participants had trouble representing the fine gradiation of force that defined each target. As a result, participants may have relied more strongly on visual feedback in this experiment than would have been the case if target force levels were more limited in scope, and therefore easier to distinguish and represent based on other sources like somatosensory and proprioceptive feedback.

Experiment 1 also left unclear exactly when motivated use of visual feedback could be used to improve performance. That is, in our task participants exert force against a dynamometer and maintain it over a fixed duration. The role of visual feedback in mediating motivated performance could vary across these stages of action implementation and maintenance.

We conducted a second experiment to address these issues. Experiment 2 was broadly similar to Experiment 1, with two changes. First, we reduced the

For Review Only

number of force targets to 3, such that each target was more clearly distinguished from the others and therefore possibly easier to represent and monitor even in the absence of visual feedback. Second, we introduced two new feedback conditions. In the LF condition, force feedback was provided only during sustained force maintenance. This meant that participants had to perform the initial force estimation without visual feedback, but could use visual feedback to monitor the consistency of their performance during each trial. In contrast, in the EF condition, force feedback was provided only until the end of the estimation period. Participants could therefore use the visual feedback to achieve target performance, but had to rely on non-visual sources like somatosensory feedback during sustained force maintenance. These additional conditions allowed us to identify precisely how visual feedback mediates the impact of motivation on force control.

For Review Only

5 Experiment 2

6 Data Analysis

As in experiment 1, we divided the analysis into two parts. First, we characterise force estimation as the averaged signed error from the target during 10 data-points (0.5 s) after the end of the estimation period (Fig. 8). We additionally calculate the consistency of this signal across trials. Second, we characterise force maintenance as the average error from the target during the maintenance period (Fig. 8), also calculating the deviation of this signal within a trial and the consistency of this signal across trials.

To analyze the data, we employ repeated-measures ANOVAs to assess the effects of exertion targets, incentives and visual feedback on force estimation and maintenance. We then used t-tests for post hoc comparisons. We define statistical significance at $p < 0.05$ and consider results with $0.05 \leq p < 0.1$ as trends.

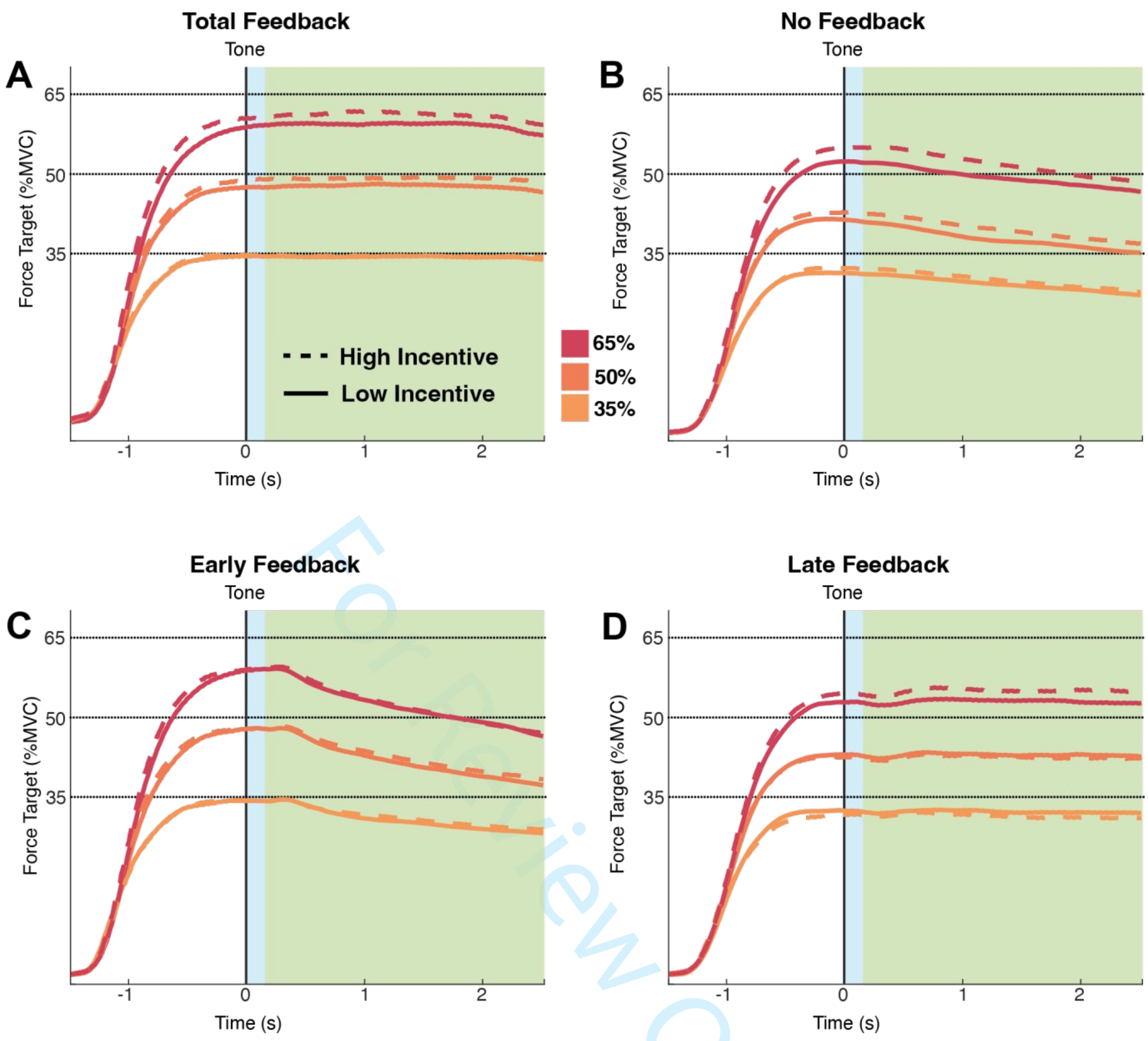

Figure 8: Force estimation and maintenance in Experiment 2 Panels A-B show the average performance across participants in the feedback and no feedback. Panels C-D show the average performance across participants in the early feedback and late feedback conditions. The section highlighted in blue was selected as the force estimation interval. The section highlighted in green was selected as the force maintenance interval.

7 Results

7.1 Initial force estimation

Initial force estimation was computed as the average distance from the target of the 10 data points after the presentation of the auditory tone indicating the end of the estimation period (See Fig. 8). Force estimation was analyzed in a three-way mixed model ANOVA with factors for exertion (3 levels), feedback (4 levels) and incentive (2 levels). The three main effects were significant (exertion: $F_{2,38}$ = 18.328, $p < 0.001$; feedback: $F_{3,57}$ = 19.349, $p < 0.001$; incentive: $F_{1,19}$ = 4.459, p

For Review Only

= 0.048), as were all two-way interactions (exertion*feedback: $F_{6,114} = 9.253$, $p < 0.001$; exertion*incentive: $F_{2,38} = 5.899$, $p = 0.005$; feedback*incentive: $F_{6,14} = 3.29$, $p = 0.027$) but the three-way interaction was not significant ($F_{6,114} = 0.789$, $p = 0.579$) (see supplementary materials SM Tables 12 to 16 for post hoc analysis).

The results are illustrated in Figure 9. As in the previous experiment, participants underestimated the target and tended to undershoot more as requested exertion increased (See Fig. 9 A-B-C-D). Error was reduced by incentive (High incentive average error 4%, Low incentive average error 5%) and reliably varied across the feedback conditions. The effect of incentive increased as a function of task exertion (See Fig. 9 E-F-G-H). This emerged across all feedback conditions, but the magnitude of the effect reliably varied as a function of feedback type, and was largest in the NF and LF conditions.

For Review Only

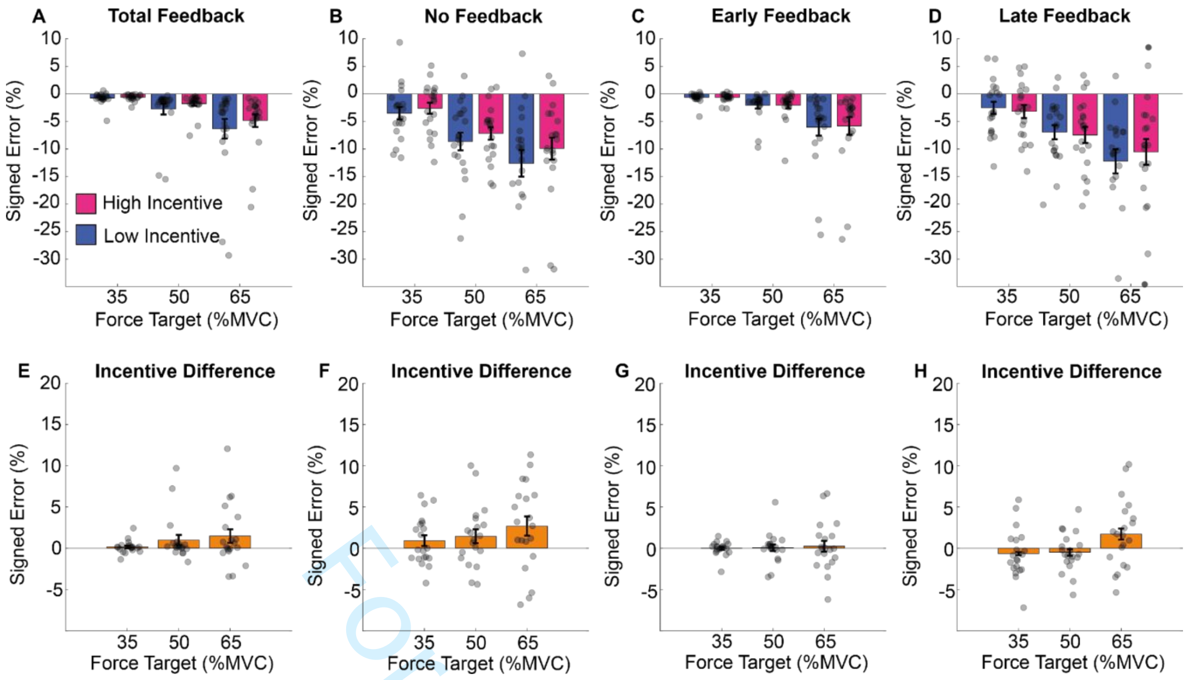

Figure 9: Mean Force Estimation in Experiment 2 Panels A-B-C-D show the mean error from the target (Y-axis) during the estimation period in the four feedback conditions (4 panels), at each force level (X-axis) and per incentive condition. Panels E-F-G-H shows the difference between high and low incentive, per force and feedback conditions.

7.1.1 Consistency across trials

Consistency was computed as the standard deviation of the mean force estimation over trials within a participant. It was analyzed in a three-way mixed model ANOVA with factors for exertion (3 levels), feedback (4 levels) and incentive (2 levels). This identified main effects of exertion ($F_{2,38} = 80.158$, $p < 0.001$) and feedback ($F_{3,57} = 78.741$, $p < 0.001$) (See supplementary Materials SM Tables 17 and 18 for post hoc analysis). No other effect reached significance (incentive: $F_{1,19} = 1.639$, $p = 0.215$; exertion*feedback: $F_{6,114} = 0.747$, $p = 0.612$; exertion*incentive: $F_{2,38} = 0.12$, $p = 0.887$; feedback*incentive: $F_{3,57} = 0.28$, $p = 0.839$; exertion*feedback*incentive: $F_{6,114} = 1.645$, $p = 0.141$).

These results are illustrated in Figure 10. Consistency decreased as a function of increasing exertion, and was poor in conditions where feedback was absent (NF) or late (LF). Incentive had no reliable impact on any pattern in this data.

For Review Only

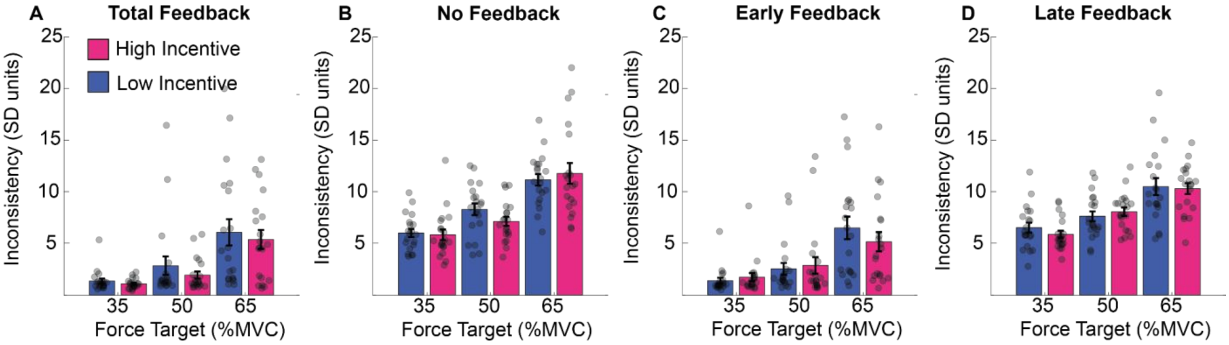

Figure 10: Consistency of Force estimation in Experiment 2 Panels A-B-C-D show the mean of the error from the target (Y-axis) during the force maintenance period the different feedback conditions (four panels), at each force level (X- axis) and per incentive condition. Force error was defined as the averaged difference at each time point between the observed force level and the current target.

7.2 Sustained Force Maintenance

Sustained force maintenance was computed as the average distance from the target of the data points after the end of the estimation period until the end of the trial. It was analyzed in a three-way mixed model ANOVA with factors for exertion (3 levels), feedback (4 levels) and incentive (2 levels). The three main effects were significant (exertion: $F_{2,38} = 33.362$, $p < 0.001$; feedback: $F_{3,57} = 31.554$, $p < 0.001$; incentive: $F_{1,19} = 5.589$, $p = 0.028$). Participants performed better in the total feedback condition, for lower levels of exertions compared to higher ones (see supplementary materials SM Table 19 and 20 for post hoc analysis) and when incentive was high (average error ~6.5%) compared to low (average error ~7.5%). The interaction of exertion by feedback was also significant ($F_{6,114} = 14.081$, $p < 0.001$), as was the interaction of exertion and incentive ($F_{2,38} = 7.001$, $p = 0.002$) (see supplementary materials SM Table 21 and 22 for post hoc analysis). No other effect reached significance (exertion*feedback*incentive: $F_{6,114} = 1.734$, $p = 0.119$; feedback*incentive: $F_{3,57} = 2.63$, $p = 0.058$).

These results are illustrated in Figure 11. Participant error increased with exertion (See Fig. 11 A-B-C-D), but performance improved as a function of both

For Review Only

feedback and incentive (See Fig. 11 A-B-C-D for the effect of feedback and panels E-F-G-H for the effect of incentive). The effect of incentive increased as a function of exertion (Fig. 11 E-F-G-H). This emerged similarly across feedback conditions.

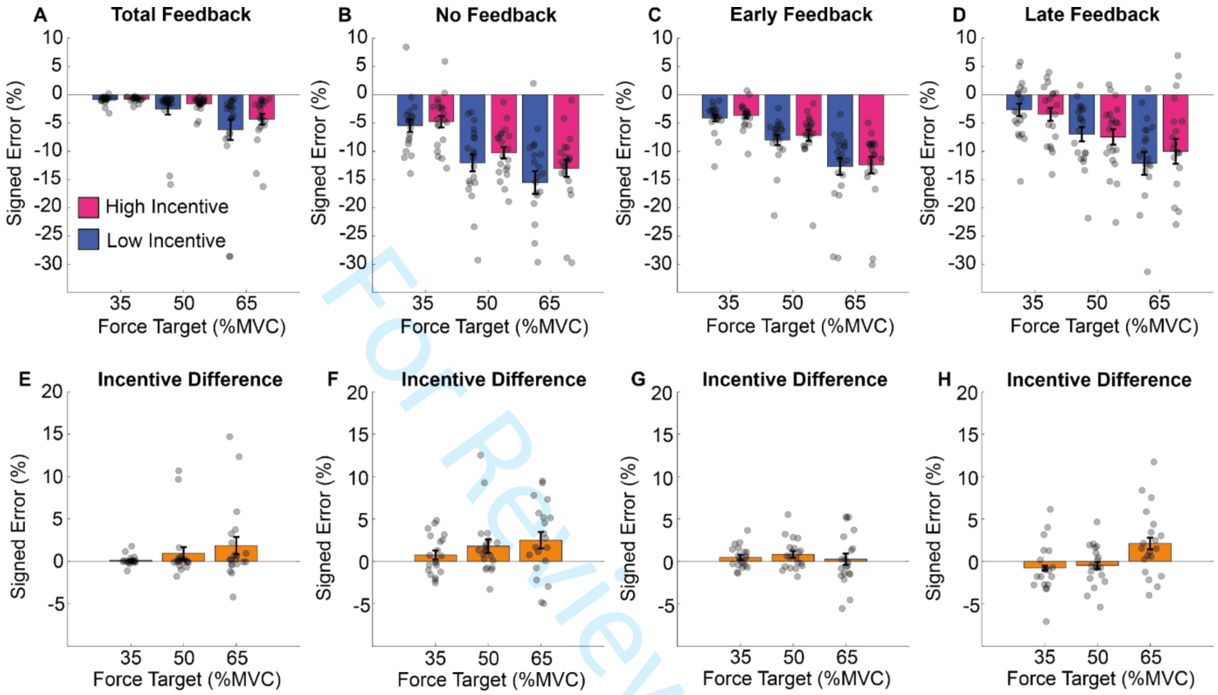

Figure 11: Error from the target in Experiment 2 Panels A-B-C-D shows for the total feedback (left panels) and for the no feedback (right panels) conditions, at each force level (X-axis) and per incentive condition the mean of the error from the target (Y-axis) during the force maintenance period. Force error was defined as the difference at each time point between observed force level and the current target. The lower panel shows the difference between high and low incentive, per force and feedback conditions.

For Review Only

7.2.1 Deviation within trials

Deviation was computed as the standard deviation of the force exerted during the maintenance period. Before performing the standard deviation, exertion data was detrended to remove the linear drift in performance. A higher standard deviation represents higher variability during the exertion. Deviation was analyzed in a three-way mixed model ANOVA with factors for exertion (3 levels), feedback (4 levels) and incentive (2 levels). The three main effects were significant (exertion: $F_{2,38} = 94.012$, $p < 0.001$; feedback: $F_{3,57} = 15.44$, $p < 0.001$; incentive: $F_{1,19} = 8.479$, $p = 0.008$). Deviation in the maintenance interval increased a.) when

feedback was absent, b.) when higher levels of exertion were requested (see supplementary materials SM Tables 23 and 24 for post hoc analysis) and c.) when incentive was low (average deviation ~1.8%) compared to high (average deviation ~1.6%).

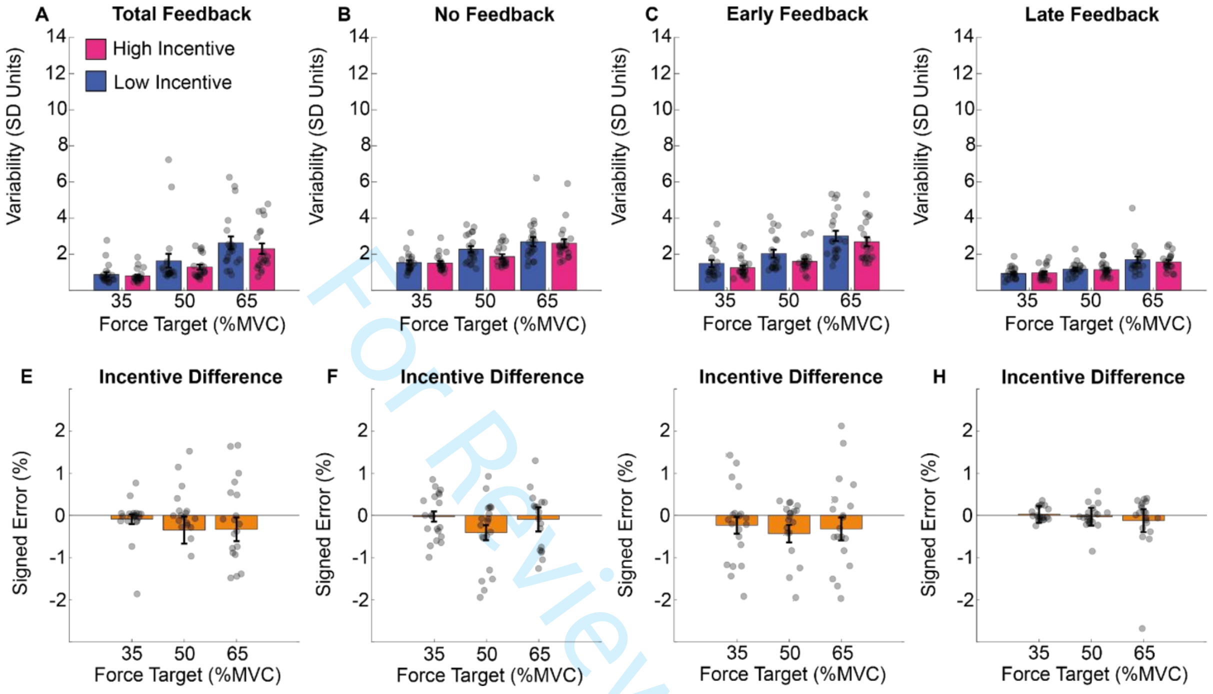

Figure 12: Deviation from the target in Experiment 2 Panels A-B-C-D shows the averaged standard deviation within trials (y-axis) for the different feedback (four panels), force (x-axis) and incentive conditions. Panels E-F- G-H show the difference between high and low incentive, per force and feedback conditions.

For Review Only

Only the exertion by feedback interaction was significant ($F_{6,114}$: 4.585, $p < 0.001$) (see supplementary materials SM Table 25 for post hoc analysis). No other effect reached significance (exertion*incentive: $F_{2,38} = 1.597$; feedback*incentive: $F_{3,57} = 0.795$, $p = 0.501$; exertion*feedback*incentive: $F_{6,114} = 0.252$, $p = 0.957$).

These results are illustrated in Figure 12. Participants' deviation from the target increased with target exertion (Fig. 12A-B-C-D). Error reduced as a function of feedback type (LF and TF; Fig. 12A-B-C-D) and incentive (Fig. 12E-F-G-H). The impact of incentive did not vary as a function of exertion requested or feedback type.

7.2.2 Consistency across trials

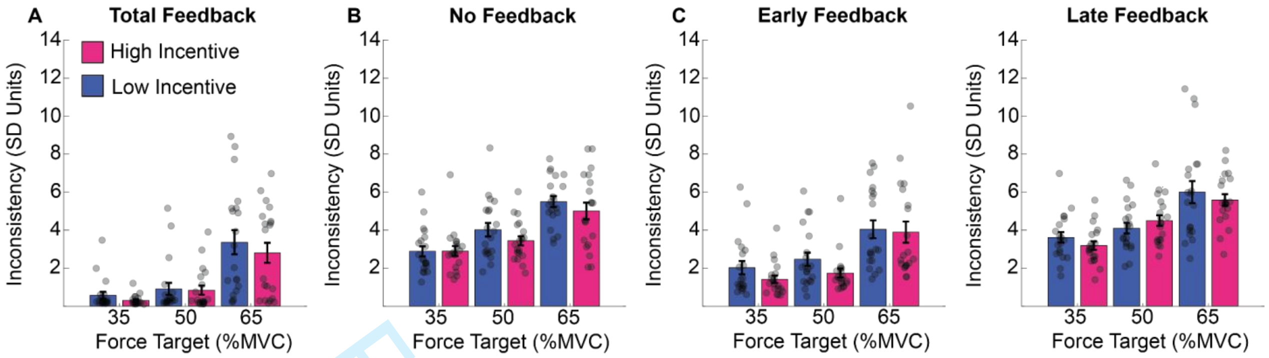

Figure 13: Force consistency in Experiment 2 Panels A-B-C-D show the results of the standard deviation across trials for the total feedback condition (left panel), for the no feedback condition (right panel), per force and incentive condition. Standard error was computed on the mean force across time points in the force maintenance period.

For Review Only

Consistency was computed as the standard deviation of the mean force exertion over trials within a participant. It was analysed in a three-way mixed model ANOVA with factors for exertion (3 levels), feedback (4 levels) and incentive (2 levels). The three-way mixed model ANOVA identified main effects of exertion ($F2{,}38 = 78.958$, $p < 0.001$) and feedback ($F3{,}57 = 59.625$, $p = 0.001$) alongside a trend toward an effect of incentive ($F1{,}19 = 867$ $p = 0.064$). Participants were more consistent when they had feedback to estimate their force (total feedback and early feedback conditions) rather than when they were estimating force (no feedback and late feedback conditions) and for lower levels of exertion compared to higher ones (see supplementary materials SM Table 26 and 27 for post hoc analysis). No other effects reached significance (exertion*feedback: $F6{,}114 = 0.611$, p

$= 0.72$; exertion*incentive: $F2{,}38 = 0.189$, $p = 0.828$; feedback*incentive: $F3{,}57 = 0.352$, $p = 0.787$; exertion*feedback*incentive: $F6{,}114 = 0.914$, $p = 0.486$).

These results are illustrated in Figure 13. Performance was more consistent

when feedback was present (ie. TF and EF conditions), but degraded as exertion increased. There was no impact of incentive on any pattern in this data.

7.3 Summary of Experiment 2

Results from Experiment 2 show a consistent effect of incentive on motor precision, regardless of the availability or quality of visual feedback. This suggests that the simplification of force targets adopted in Experiment 2 allowed participants to represent targets based solely on somatosensory and proprioceptive information. As such, they were able to monitor this information and optimize behaviour in high incentive conditions.

8 Discussion

Achieving precise motor control necessitates the integration of sensory input with internal representation to execute movement plans effectively (Cappadocia, Monaco, Chen, Blohm, & Crawford, 2017; Velji-Ibrahim, Crawford, Cattaneo, & Monaco, 2022). Subsequently, newly generated sensory feedback fine-tunes movement online (Crevecoeur, Cluff, & Scott, 2014; Turella, Rumiati, & Lingnau, 2020), with visual and proprioceptive information playing pivotal roles in this process (Sartin, Ranzini, Scarpazza, & Monaco, 2022; Monaco et al., 2010; Filimon, Nelson, Huang, & Sereno, 2009; Monaco et al., 2006). The two experiments reported here demonstrate the important role of visual feedback in mediating the effect of motivation on force generation accuracy and precision. In Experiment 1, we found that the impact of incentive motivation was entirely contingent on the provision of visual performance feedback. In Experiment 2, where performance targets were easier to distinguish from proprioceptive and somatosensory feedback, the benefit of motivation emerged in both total feedback and no feedback conditions.

For Review Only

We interpret this as evidence that motivation can impact difficult, fine motor performance even when visual performance feedback is not available. This may occur through a direct impact that decreases noise in the motor system, or through an indirect influence on participant monitoring of proprioceptive and somatosensory performance feedback.

8.1 Effect of Visual Feedback and exertion targets

Consistent with previous literature (Limonta et al., 2015; Noble et al., 2013; Vaillancourt et al., 2003; Slifkin et al., 2000; Baweja et al., 2010), our results underscore the critical role of visual feedback in monitoring force control accuracy and reducing variability, particularly in circumstances where target performance is subtle. Additionally, our analyses unveil a significant impact of exertion on force production, with increased demands leading to greater variability and deviations from the target. Returning to the example described in the introduction, our waiter is in a situation where force targets vary as a function of what drinks have been placed on the tray and of the physics of the waiter's navigation through the restaurant. Under these circumstances, he will struggle to maintain balance and control of his tray without visual feedback.

8.2 Roles of feedback in force estimation and maintenance

In our second experiment, we introduced two novel feedback conditions: Early Feedback and Late Feedback. These conditions allowed us to compare the distinct effects of feedback on force estimation and force maintenance. In the Early Feedback condition, participants received feedback during the force estimation phase but not during the force maintenance phase. Participants' force estimation performance mirrored that observed in the feedback condition. However, once the feedback was withdrawn during the maintenance phase, the motor decay and the

For Review Only

effect of incentives on performance did not significantly differ from the no feedback condition. Conversely, in the Late Feedback condition, participants received no feedback during the force estimation phase, relying entirely on their internal representation of the target force. Feedback was then introduced during the maintenance period. Here, participants' estimation resembled that of the no feedback condition. However, once feedback was introduced during the maintenance phase, performance mirrored the one observed in the feedback condition, with participants demonstrating reduced variability.

8.3 Interaction with Monetary Incentives

Incentive influences force production and this is evident in its ability to boost motivation, stimulate robust muscle contractions, and influence the exertion/rest trade-off (Klein-Flügge et al., 2016; Croxson et al., 2009; Le Bouc et al., 2016; Zénon et al., 2016; Pessiglione et al., 2007; Oudiette et al., 2019; Meyniel et al., 2013; Müller et al., 2021). Expanding on these findings, our study delved into the role of incentives in fine motor control. We observed an interaction between monetary incentives and exertion targets that particularly affects force accuracy and variability. While incentives positively impacted force control accuracy across all exertion levels, this effect was most pronounced under high level exertion. Participants demonstrated enhanced accuracy and reduced variability in force production when motivated by higher monetary incentives. This suggests that incentive plays a crucial role in reducing errors in force production, especially when the task demands are high. In easier conditions, the motor system might be able to perform adequately without a strong motivational push. However, when the task becomes more challenging and errors become more likely, incentives appear to act as a facilitator, promoting greater focus, enhanced accuracy, and

For Review Only

reduced variability in force production (Codol, Holland, Manohar, & Galea, 2020).

8.4 Role of Incentives in Feedback Modulation

Crucially, our findings indicate that the influence of incentives on force control depends on the ability to reliably monitor performance. In Experiment 1, incentives exerted a strong effect on force accuracy and consistency, especially under high level exertion requests. However, when visual feedback was absent, this effect disappeared. This suggests that incentive effects on motor precision were mediated by the availability of reliable visual feedback (Sporn, Chen, & Galea, 2022; Codol et al., 2023).

The second experiment presented a contrasting scenario. Here, participants formed a clear internal representation of the target force without relying on visual feedback. Interestingly, even in the absence of visual feedback, incentives continued to influence motor precision, particularly in terms of force estimation. This suggests that when a clear internal representation exists, incentives can exert a more direct effect on the motor control system itself, potentially influencing initial force generation and estimation before sensory feedback comes into play.

As noted above, the effect of motivation on performance in the absence of visual feedback could reflect a direct influence on the motor signal itself, to reduce internal noise in this system, or could act through a potentiation of how proprioceptive and somatosensory feedback is monitored by the participant. Our results show that, when visual feedback is absent, incentives have a particular impact on initial force estimation rather than force maintenance, and this is consistent with the idea of a direct effect on motor control. However, it is also likely that enhanced monitoring of proprioceptive and somatosensory feedback

For Review Only

plays a role here, and identifying the precise involvement of each mechanism will require further experimentation.

In summary, our results demonstrate that motivational effects on fine motor control rely strongly on enhanced monitoring of visual feedback. This is the case in the common scenario where performance targets differ subtly, and are therefore difficult to represent in terms of proprioception and somatosensation. However, when targets are more easily distinguished in these terms, motivation will benefit performance even in the absence of visual feedback. This was further clarified in the second experiment with the introduction of two feedback conditions where feedback was manipulated in either the force estimation or the force maintenance. Visual feedback therefore plays an important role in mediating motivational effects on fine motor performance, but these effects can be instantiated more directly when levels of target performance are unambiguous and easily represented.

For Review Only

Declarations

Funding: ‘Not applicable’

Conflict of interest: The authors declare no competing interests.

Ethics approval and Consent to participate: All participants gave informed written consent and the study procedure was approved by the local institutional review board of the University of Trento.

Consent for publications: All authors have given their consent for publication.

Data and Code Availability Statement: The data and code supporting the findings of this study are available on Hub at the following link: https://github.com/Nich0Me/ForceFeedbackIncentive.

Aknowledgements

We thank Thibaud Griessinger, Elio Balestrieri, Damiano Grignolio and Nadege Bault for their assistance and discussion of the project. CH is supported by the European Research Council under the European Union Horizon 2020 Research and Innovation Program (804360).

References

Abolins, V., & Latash, M. L. (2022). Unintentional force drifts as consequences of indirect force control with spatial referent coordinates. Neuroscience, 481, 156–165.

Abolins, V., Ormanis, J., & Latash, M. L. (2023). Unintentional drifts in performance during one-hand and two-hand finger force production. Experimental Brain Research, 241 (3), 699–712.

Adkins, T. J., Gary, B. S., & Lee, T. G. (2021). Interactive effects of incentive value and valence on the performance of discrete action sequences. Scientific Reports, 11 (1), 1–12.

Apps, M. A., Grima, L. L., Manohar, S., & Husain, M. (2015). The role of cognitive effort in subjective reward devaluation and risky decision-making. Scientific reports, 5 (1), 16880.

Baweja, H. S., Kennedy, D. M., Vu, J., Vaillancourt, D. E., & Christou, E. A. (2010). Greater amount of visual feedback decreases force variability by reducing force oscillations from 0–1 and 3–7 hz. European journal of applied physiology, 108 , 935–943.

Brainard, D. H. (1997). The psychophysics toolbox. Spatial Vision, 10 , 433-436.

Cappadocia, D. C., Monaco, S., Chen, Y., Blohm, G., & Crawford, J. D. (2017). Temporal evolution of target representation, movement direction planning, and reach execution in occipital–parietal– frontal cortex: an fmri study. Cerebral Cortex, 27 (11), 5242–5260.

Codol, O., Holland, P. J., Manohar, S. G., & Galea, J. M. (2020). Reward-based improvements in motor control are driven by multiple error-reducing mechanisms. Journal of Neuroscience, 40 (18), 3604–3620.

Codol, O., Kashefi, M., Forgaard, C. J., Galea, J. M., Pruszynski, J. A., & Gribble, P. L. (2023). Sensorimotor feedback loops are selectively sensitive to reward. Elife, 12, e81325.

Crevecoeur, F., Cluff, T., & Scott, S. H. (2014). Computational approaches for goal-directed movement planning and execution. The Cognitive Neurosciences, 461, 475.

Croxson, P. L., Walton, M. E., O'Reilly, J. X., Behrens, T. E., & Rushworth, M. F. (2009). Effort-based cost-benefit valuation and the human brain. Journal of Neuroscience, 29 (14), 4531–4541.

Filimon, F., Nelson, J. D., Huang, R.-S., & Sereno, M. I. (2009). Multiple parietal reach regions in humans: cortical representations for visual and proprioceptive feedback during online reaching. Journal of Neuroscience, 29 (9), 2961–2971.

Goodale, M. A., & Milner, A. D. (1992). Separate visual pathways for perception and action. Trends in neurosciences, 15 (1), 20–25.

Klein-Flügge, M. C., Kennerley, S. W., Friston, K., & Bestmann, S. (2016). Neural signatures of value comparison in human cingulate cortex during decisions requiring an effort-reward trade-off. Journal of Neuroscience, 36 (39), 10002–10015.

Le Bouc, R., Rigoux, L., Schmidt, L., Degos, B., Welter, M.-L., Vidailhet, M., . . . Pessiglione, M. (2016). Computational dissection of dopamine motor and motivational functions in humans. Journal of Neuroscience, 36 (25), 6623–6633.

Limonta, E., Rampichini, S., Cè, E., & Esposito, F. (2015). Effects of visual feedback absence on force control during isometric contraction. European journal of applied physiology, 115, 507–519.

Manohar, S. G., Chong, T. T.-J., Apps, M. A., Batla, A., Stamelou, M., Jarman, P. R., . . . Husain, M. (2015). Reward pays the cost of noise reduction in motor and cognitive control. Current Biology, 25 (13), 1707–1716.

Mayhew, S. D., Porcaro, C., Tecchio, F., & Bagshaw, A. P. (2017). fmri characterisation of widespread brain networks relevant for behavioural variability in fine hand motor control with and without visual feedback. Neuroimage, 148 , 330–342.

Meyniel, F., Sergent, C., Rigoux, L., Daunizeau, J., & Pessiglione, M. (2013). Neurocomputational account of how the human brain decides when to have a break. Proceedings of the National Academy of Sciences, 110 (7), 2641–2646.

For Review Only

Milner, A. D., & Goodale, M. A. (2008). Two visual systems re-viewed. Neuropsychologia, 46 (3), 774–785.
Monaco, S., Fattori, P., Galletti, C., Goodale, M. A., Króliczak, G., Quinlan, D., & Culham, J. C. (2006). The contribution of visual and proprioceptive information to the precision of reaching movements. Journal of Vision, 6 (6), 397–397.
Monaco, S., Króliczak, G., Quinlan, D. J., Fattori, P., Galletti, C., Goodale, M. A., & Culham, J. C. (2010). Contribution of visual and proprioceptive information to the precision of reaching movements. Experimental brain research, 202, 15–32.
Müller, T., Klein-Flügge, M. C., Manohar, S. G., Husain, M., & Apps, M. A. (2021). Neural and computational mechanisms of momentary fatigue and persistence in effort-based choice. Nature Communications, 12 (1), 4593.
Noble, J. W., Eng, J. J., & Boyd, L. A. (2013). Effect of visual feedback on brain activation during motor tasks: an fMRI study. Motor Control, 17 (3), 298–312.
Oudiette, D., Vinckier, F., Bioud, E., & Pessiglione, M. (2019). A pavlovian account for paradoxical effects of motivation on controlling response vigour. Scientific Reports, 9 (1), 1–13.
Pessiglione, M., Schmidt, L., Draganski, B., Kalisch, R., Lau, H., Dolan, R. J., & Frith, C. D. (2007). How the brain translates money into force: a neuroimaging study of subliminal motivation. science, 316 (5826), 904–906.
Sartin, S., Ranzini, M., Scarpazza, C., & Monaco, S. (2022). Cortical areas involved in grasping and reaching actions with and without visual information: an ale meta-analysis of neuroimaging studies. Current Research in Neurobiology, 100070.
Slifkin, A. B., & Newell, K. M. (1999). Noise, information transmission, and force variability. Journal of Experimental Psychology: Human Perception and Performance, 25(3), 837
Slifkin, A. B., Vaillancourt, D. E., & Newell, K. M. (2000). Intermittency in the control of continuous force production. Journal of Neurophysiology, 84 (4), 1708–1718.
Sporn, S., Chen, X., & Galea, J. M. (2022). The dissociable effects of reward on sequential motor behavior. Journal of Neurophysiology, 128 (1), 86–104.
Turella, L., Rumiati, R., & Lingnau, A. (2020). Hierarchical action encoding within the human brain.
Cerebral cortex, 30 (5), 2924–2938.
Vaillancourt, D. E., & Russell, D. M. (2002). Temporal capacity of short-term visuomotor memory in continuous force production. Experimental brain research, 145, 275–285.
Vaillancourt, D. E., Slifkin, A. B., & Newell, K. M. (2001). Intermittency in the visual control of force in Parkinson's disease. Experimental Brain Research, 138, 118–127.
Vaillancourt, D. E., Thulborn, K. R., & Corcos, D. M. (2003). Neural basis for the processes that underlie visually guided and internally guided force control in humans. Journal of Neurophysiology, 90 (5), 3330–3340.
Velji-Ibrahim, J., Crawford, J. D., Cattaneo, L., & Monaco, S. (2022). Action planning modulates the representation of object features in human fronto-parietal and occipital cortex. European Journal of Neuroscience, 56 (6), 4803–4818.
Whittier, T. T., Patrick, C. M., & Fling, B. W. (2023). Somatosensory information in skilled motor performance: A narrative review. Journal of Motor Behavior, 55 (5), 453–474.
Zénon, A., Devesse, S., & Olivier, E. (2016). Dopamine manipulation affects response vigor independently of opportunity cost. Journal of Neuroscience, 36 (37), 9516–95.

For Review Only

For Review Only
